# RNAs anchoring replication complex control initiation and firing of DNA replication

**DOI:** 10.21203/rs.3.rs-5723221/v1

**Published:** 2025-01-28

**Authors:** Simone Ummarino, Larysa Poluben, Alex K. Ebralidze, Ida Autiero, Yanzhou Zhang, Theodore Paniza, Madhura Deshpande, Lucrezia Rinaldi, Johnathan D. Lee, Mahmoud A. Bassal, Bon Q. Trinh, Steven P. Balk, Robert Flaumenhaft, Jeannine Gerhardt, Sergei M. Mirkin, Daniel G. Tenen, Annalisa Di Ruscio

**Affiliations:** 1 Harvard Medical School Initiative for RNA Medicine, Harvard Medical School, Boston, MA, 02115, USA; 2 Harvard Stem Cell Institute, Harvard Medical School, Boston, MA, 02115, USA; 3 Cancer Research Institute, Beth Israel Deaconess Medical Center, Boston, 330 Brookline Avenue Boston, MA 02215, USA; 4 Beth Israel Deaconess Medical Center, Department of Medicine, Division of Hematology-Oncology, Boston, MA, 02115, USA; 5 Institute of Biostructures and Bioimaging, CNR, Naples, 80100, Italy; 6 Center for Reproductive Medicine, Weill Cornell Medical College, New York, NY 10065, USA; 7 Cancer Science Institute of Singapore, National University of Singapore, 117456, Singapore; 8 University of Virginia; 9 Beth Israel Deaconess Medical Center, Division of Hemostasis and Thrombosis, Harvard Medical School, Boston, MA, 02115, USA; 10 Department of Biology, Tufts University, Medford, MA 02155, USA; 11 University of Eastern Piedmont, Department of Translational Medicine, Novara, 28100, Italy; 12 These two authors equally contributed to the work.; 13 These two authors equally contributed to the work.

## Abstract

Coordinated initiation of DNA replication is essential to ensure efficient and timely DNA synthesis. Yet, molecular mechanism describing how replication initiation is coordinated in eukaryotic cells is not completely understood. Herein, we present data demonstrating a novel feature of RNAs transcribed in the proximity of actively replicating gene loci. We show that RN*A*s a*NC*horing *OR*C1 (*ANCORs*) to the histone variant H2A.Z are licensors of the DNA replication process. This *ANCOR*-H2A.Z interaction is essential for cells to initiate duplication of their genetic material. Widespread and *locus*-specific perturbations of these transcripts correlate with anomalous replication patterns and a notable loss of the H2A.Z replicative marker at the origin site.

Collectively, we present a previously undescribed RNA-mediated mechanism that is associated with the generation of active replication origins in eukaryotic cells. Our findings delineate a strategy to modulate the origins of replication in human cells at a local and global level, with potentially broad biomedical implications.

## INTRODUCTION

In human cells, more than 6 billion base pairs of DNA need to be replicated and packaged into chromatin at every cell division cycle. Disruptions to this process can lead to mutations and chromosomal aberrations with consequent effects on genomic integrity and the epigenome, potentially leading to genetic diseases and cancer ^1^. Bacteria have a unique, well-defined origin of replication, whereas eukaryotes have multiple origins of replication ^2^. In yeast, conserved autonomously replicating sequences (ARS) have been thoroughly characterized ^3^. By contrast, in mammals, replication origins are not conserved among cell types or tissues, and only a handful of them have been characterized ^4–6^. At present, the molecular mechanisms controlling the distribution and activation of replication origins across diverse cell types and developmental stages remain relatively unexplored.

DNA replication timing (RT) defined as the temporal order of chromosomal segments replicated during the S phase in the cell cycle is cell-type specific. Human cell differentiation involves reproducible changes in RT. In fact, RT is disrupted in malignant transformation, and comparisons of RT profiles between normal and diseased tissues led to the identification of replication defects at specific gene loci, e.g., *c-MYC, FXN1* and *ROR1*, that would not be detected by standard transcriptome analyses ^7–9^.

Studies of budding yeast and mammals have established that origin recognition is mediated by a hexameric protein complex, termed the Origin Recognition Complex (ORC)^10–13^. Recent developments in click chemistry-based approaches have facilitated the identification of multiple replication origins and confirmed the role of Origin Recognition Complex Subunit 1 (ORC1) in origin selection and RT ^14^. Further, CryoEM structural analyses have suggested the existence of a pre-licensing step wherein the interaction of ORC1 with the preassembled ORC2–5 complex promotes the opening of the ORC core, facilitating its DNA binding ^15^. Yet, despite multiple efforts, no consensus sequence motif has emerged from the analyses of ORC1 DNA binding sites. Intriguingly, enrichment at enhancers and promoters marked by activating histone chromatin modifications have been reported for ORC1 ^16,17^. Altogether, these findings suggest that the plasticity of mammalian DNA replication programs could result from specific epigenetic features that modulate the selection and activation of the replication origins.

H. Long et al. (2020) have proposed a model wherein the histone variant H2A.Z, the major H2A variant associated with gene activation, recruits ORC1 at early replication origins in an H4K20me2-dependent manner ^18^. Of note, this model does not address what leads to the enrichment of H2A.Z and coordinated ORC1 engagement at early G1/S phase replicative origins. In this study, we unveil the role of previously neglected participants, namely, RN***A***s a***NC***horing ***OR***C1 (***ANCORs***) as licensors of mammalian replication origins, and describe a mechanism by which *ANCORs* engage ORC1 genome-wide while enabling binding of the histone variant H2A.Z ^19–21^. Importantly, perturbation of the *ANCOR-H2A.Z* interaction results in parallel changes in DNA replication origins. Our findings uncover a previously undescribed RNA-mediated mechanism linking genetic and epigenetic features of mammalian replication origins and define an uncharted mechanism to control RT in human cells.

## RESULTS

### *c-MYC G1/*S phase transcribed RNAs are implicated in c*-MYC* origin formation.

**Figure 1|:**
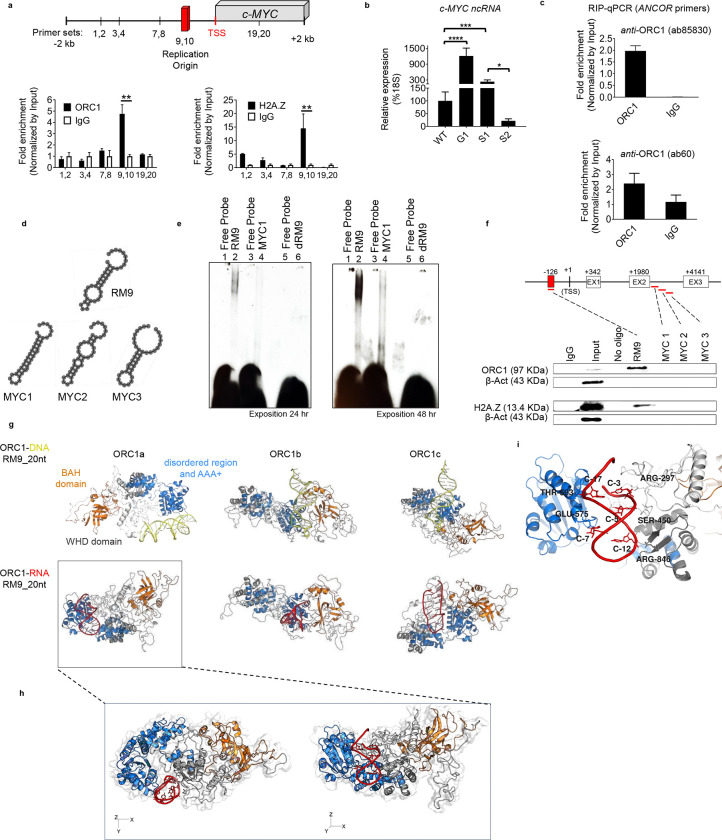
c-*MYC G1/*S phase RNAs interact with ORC1. **a.** (**Upper panel**). Schematic of the *c-MYC* locus and respective primer sets indicated by numbers. The red bar indicates the *c-MYC* replication origin (region 9–10). (**Bottom panel**) ORC1 and H2A.Z enrichment at the *c-MYC* origin detected by chromatin immunoprecipitation (ChIP). The qPCR analysis was performed using distinct sets of primers designed as depicted in the schematic diagram provided above. The bars indicate the mean ± S.D of three independent experiments (n=3), Student’s t-test, **: p value 0.0085 (ORC1) and **: p value 0.0055 (H2A.Z); **b** Expression levels of RNAs originating within the promoter of c-MYC, G1 and later stages of the S phase: S1 (2hr), S2 (5hr). The bars represent the mean ± S.D of three independent experiments (n=3), Student’s t-test, *: p value <0.0001 (G1 vs WT), p value <0.0001 (S1 vs WT) and p value 0.00189 (S1 vs S2) **c.** RNA immunoprecipitation (RIP) qRT-PCR performed using two separate antibodies against human ORC1 (ab85830 and ab60). The association between c-MYC S-Phase RNAs anchoring ORC1 – ANCOR is shown **d-f.** *In vitro* RNA/DNA electrophoresis mobility shift assay (EMSA) and RNA pull-down assays. **d.** RNA secondary structures of the RNA probes corresponding to the *c-MYC* origin (RM9) and unrelated to the origin (MYC1, MYC2, and MYC3) calculated using RNAfold; **e.** RNA EMSA showing the preferential binding of ORC1 to RM9 (RNA) *versus* the unrelated MYC1 sequence and the homologous DNA sequence (dRM9); **f.** RNA pull down assay. Bound proteins were purified on streptavidin beads and immunoblotted with antibodies against ORC1 and H2A.Z. The experiment was repeated twice with similar results; **g.** Molecular dynamics simulations: frontal views of the representative structures of ORC1a, ORC1b and ORC1c states in complex with either dRM9 (DNA) or RM9 (RNA). RNA and DNA structures were derived using a RMSD-based clustering approach in the last half of simulation time. All molecules are color-coded as follows: orange: ORC1-BAH domain (residues 1–200); marine: ORC1 disordered region and AAA+ domain (residues 201–782); gray: orc1-WHD domain (residues 783–861); red and yellow: RM9 and dRM9, respectively; **h.** The figure shows a zoomed-in view of both the top and frontal perspectives, illustrating the preferred binding state of the ORC1-RM9 complex; i. Zoomed-in view showing the ORC1 amino-acid residues interacting with RM9.

Herein, we investigated the contribution of G1/S-phase transcribed RNAs in the initiation of early replication origins. The rationale was twofold: 1) the coordination by cell type- and phase-specific RNAs would reconcile the multiple replication origins observed across different cells, and 2) the colocalization of H2A.Z and ORC1 at early replication origins would align with recent findings linking G1/S phase transcribed RNAs to the deposition and modification of the histone variant H2A.Z ^22^. First, we surveyed the *c-MYC* locus, the origin of which has been extensively studied ^4,6^. To examine the enrichment of ORC1 and H2A.Z at the *c-MYC* origin ^6^, hereafter referred as region 9–10 (Figure 1 a), we performed chromatin immunoprecipitation (ChIP) with validated antibodies, followed by quantitative (q)PCR (Figure 1a, Supplementary Figure 1a). We detected 4.7- and 14.5-fold enrichment within region 9–10 for ORC1 and H2A.Z, respectively, suggesting the presence of both proteins at the target locus (Figure 1a). Next, we probed the nuclear fraction expression of RNAs arising from the promoter and encompassing the *c-MYC* origin during the G1 phase and over the release from the S phase: S1 (2 hr), S2 (5 hr) (Figure 1b, Supplementary Figure 1b,c) ^23^. The highest expression levels of *c-MYC* promoter and origin transcripts were detected in the G1 phase, followed by the early S phase (S1), with a marked decline in later S phase stages (S2), as compared to the unsynchronized cells (Figure 1b) ^22,24^.

The precise mechanisms guiding the localization of human (h)ORC1 on chromatin and its replication function remain poorly understood ^25^. RNAse A treatment impairs ORC1 recruitment to the Epstein–Barr virus plasmid replication origin *in vitro* ^25^, as well as the interaction between telomeric repeat-binding factor 2 (TRF2) and ORC1 ^26^. Recent studies have further indicated preferential binding of ORC1 to G-rich RNA or single-stranded DNA sequences *in vitro* ^27^, indicating a potential contribution of RNA to ORC1 recruitment. Thus, we explored the possibility of an RNA-dependent mechanism coordinating the initiation and chromatin status at replication origins in eukaryotic cells. To determine whether ORC1 could physically associate with the ***c-MYC G1/*S** phase transcribed **RNAs**, we performed RNA immunoprecipitation (RIP) with two different ORC1 antibodies (Figure 1c). We observed enrichment of *c-MYC* RNAs in the ORC1-RNA precipitates and termed these transcripts *c-MYC ANCORs* (RN***A***s a***NC***horing ***OR***C1).

To study the molecular properties of the RNA-ORC1 interaction *in vitro*, we performed an RNA/DNA electrophoresis mobility shift assay (EMSA) and RNA pull-down assay with K562 cell extracts (Figures 1d–f, Supplementary Figure 1d). The RNA oligonucleotides were selected based upon: (1) the proximity to the *c-MYC* origin and higher CG content (RM9) as well as (2) the distance from the *c-MYC* origin and the lower CG content (MYC1,2,3). DNA oligonucleotides (dRM9) corresponding to the RM9 sequence were also analyzed to compare the RNA and DNA interactions with ORC1. RNA-ORC1 complex formation was observed with RM9 (Figure 1e, left and middle panels of Supplementary Figure 1d). By contrast, no binding with the homologous DNA oligonucleotide was detected, suggesting a non-preferential interaction of ORC1 with DNA as compared to RNA (Figure 1e, left and middle panels of Supplementary Figure 1d). RNA oligonucleotides unrelated to the *c-MYC* origin failed to produce a shift by RNA EMSA (Figure 1e, left and middle panels of Supplementary Figure 1d) and did not produce ORC1 enrichment in the RNA pull-down assay (Figure 1f), suggesting binding specificity between RM9-ORC1. We confirmed the presence of H2A.Z in the RM9 precipitates by RNA pull-down, indicating the presence of both ORC1 and H2A.Z in the complex (Figure 1f). Consistently, the interaction between H2A.Z and RM9 but not MYC1 was also detected by EMSA (right panel of Supplementary Figure 1d), supporting the dual role of RNA in coordinating the localization of ORC1 while enabling binding to the histone variant H2A.Z ^22^.

Next, we sought to analyze *in silico* the interaction between (h)ORC1 and *c-MYC ANCOR* with respect to the equivalent DNA sequence to determine the structural and dynamic features of ORC1-RNA complexing. The interaction between ORC1 and RNA (RM9) and the respective DNA sequence (dRM9) was modeled using computational approaches. Three apo ORC1 states (ORC1a, ORC1b and ORC1c) were considered equally probable conformations targeted by the sequence (Figure 1g, Supplementary Figure 1e). These states were derived from three distinct molecular dynamics (MD) trajectories performed starting from the AlphaFold ORC1 full-length model with the application of diverse velocities to maximize the exploration of the protein conformational ensemble. The considered states mainly differ from the relative orientation between the N- and C- ORC1 regions and that of the bromo adjacent homology domain (BAH domain), consistent with the prevalence of disordered and flexible residue patches between them. Both RM9 and dRM9 were docked against each of the three ORC1 states, and the obtained complexes were subjected to extended MD simulations to assess the reliability of the interaction (Supplementary Figure 1f). Five out of six of the ORC1-RNA complexes showed a deviation from the initial binding mode. However, the ORC1a-RM9 complex revealed a conserved protein-RNA interface and no notable structural rearrangements of either molecular entity. These findings indicate that RM9 efficiently targets ORC1 at the specific binding site exhibited by the ORC1a conformation. The simulation revealed the smallest perturbations from the initial state, as reflected by the lowest mean value of the root mean squared deviation (RMSD) profiles with the respect to the starting simulation state (Supplementary Figure 1f). Indeed, RM9 fits within the pocket embraced by the disordered region of ORC1 and the AAA+ domain, otherwise inaccessible within the 1b and 1c conformation (Figures 1g, h). Along with the simulation (Tables S1–S2), the ORC1-RM9 hydrogen indicates that RM9 mainly connects to ORC1a through the ARG297, SER450, GLU575, THR593 and ARG846 residues, with occurrences greater than the 70% among the frames of the last 250 ns of trajectory (Figure 1i, Supplementary Figure 1g and Table S2). No significant rearrangements of the local structure of the protein were induced by RM9 targeting. Nevertheless, during the simulation, BAH approached the other protein domains (Supplementary Figure 1h), suggesting that RM9 likely plays a role in favoring a specific ORC1 conformational equilibrium. We did not observe an effective binding mode in the ORC1a-dRM9 simulation as shown by RM9. Importantly, the simulations evidenced that the DNA binding is facilitated by the involvement of the BAH domain in the interaction. This is consistent with the established knowledge that the BAH domain interacts with DNA/chromatin ^28–30^. In contrast, the most efficient RM9 binding mode resulted from the exposure of a specific pocket in the ORC1a conformation, wherein the BAH domain was displaced from the interacting region (Figure 1g, h and Supplementary Table S1, S2).

Collectively, these analyses demonstrate distinct RNA and DNA binding modalities to ORC1, supporting the results obtained by EMSA and RNA pull-down (Figures 1 d–f, Supplementary Figure 1d). In summary, these results denote colocalization of H2A.Z and ORC1 mediated by *c-MYC ANCOR* and suggest preferential binding of ORC1 to RNA over DNA both *in vitro* and *in silico.*

### *c-MYC ANCORs* control the replication process at the *c-MYC* locus.

**Figure 2|:**
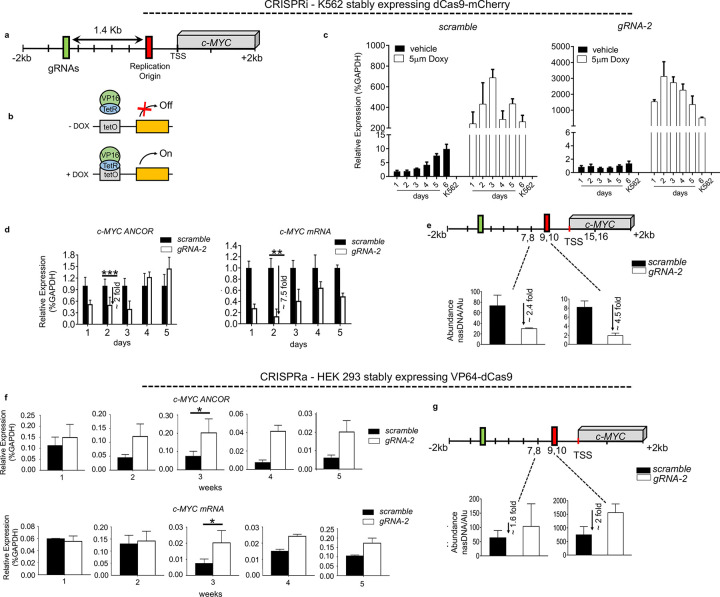
*c-MYC ANCOR* levels regulate DNA replication at the *c-MYC locus*. **a**. Outline of the *c-MYC locus* is shown, indicating the position of the guide RNA-2 (gRNA-2) sequence, located 1.4 kilobases (kb) upstream of the *c-MYC* replication origin; **b**. Schematic representation of the doxycycline-responsive promoter (TET-ON) employed to clone gRNA-2 for the inducible CRISPR interference (CRISPRi) in K562 stably expressing dCas9-mCherry; **c**. Time-course analysis by quantitative reverse transcription-PCR (qRT-PCR) of scrambled control and gRNA-2 expression following a single 5 mM doxycycline addition; **d**. qRT-PCR analysis of *c-MYC ANCOR* and *c-MYC* expression over 6 days upon induction of gRNA-2 and scramble control with a single doxycycline addition. The mean ± S.D. of three independent experiments (n=3) is shown, Student’s t-test, ***: p value 0.0002 (*c-MYC ANCOR*) and **: p value 0.0021 (*c-MYC mRNA*); **e**. Quantification of nascent DNA (nasDNA) abundance at the *c-MYC* origin shows a significant decrease, 2.4- and 4.5-fold, at regions 7–8 and 9–10, respectively, encompassing the *c-MYC* origin in response to the suppression of *c-MYC ANCOR*. Measurements were taken at day 3 following induction of gRNA-2 and scramble control; **f-g**. CRISPR activator in HEK 293. **f**. gRNA-2 introduced in HEK 293 constitutively expressing VP64-dCas9 results in upregulation of *c-MYC ANCOR* and *c-MYC* mRNA. The mean ± S.D. of three independent experiments (n=3) is shown, Student’s t-test, *: p value 0.0161 (*c-MYC ANCOR*) and **: p value 0.0015 (*c-MYC mRNA*); **g**. Activation of *c-MYC ANCOR* was associated with an increase in newly synthesized DNA strands of 1.6- and 2-fold within regions 7–8 and 9–10, respectively, surrounding the *c-MYC* origin.

To assess whether alterations in the transcriptional levels of c*-MYC ANCORs* could impact the integrity of the replication process at the *c-MYC* origins, we performed both CRISPR-mediated loss- and gain-of-function experiments. Loss-of-function was achieved by repressing *c-MYC ANCORs* using CRISPR interference (CRISPRi, CRISPR-dCAS9 system)^31^ in the K562 cell line. Three single guide RNAs (gRNA1–2), targeting a region located 1.4kb upstream of the *c-MYC* origin, were constitutively induced, along with the scramble control, in K562 cells stably expressing dCas9-mCherry (K562 dCas9-mCherry) (Supplementary Figure 2a,b). The gRNAs were specifically designed to interfere with *c-MYC ANCOR* transcription without impairing the integrity of the replication origin. gRNA-2 led to lasting downregulation of *c-MYC* ANCOR, followed by a reduction in *c-MYC* mRNA levels (Supplementary Figure 2c). To rule out a potential off-target effect of CRISPRi on *c-MYC* expression, wild-type K562 cells were treated with FANA antisense oligonucleotides (ASOs) exclusively targeting *c-MYC ANCOR*. The same decrease in *c-MYC* expression was confirmed upon a reduction in *c-MYC ANCOR* (Supplementary Figure 2d). Hence, gRNA-2 was selected for all downstream experimental analyses. To control the expression of the gRNAs, gRNA-2 and the respective scramble sequence were cloned under a Tet-inducible promoter and delivered into K562-dCas9-mCherry cells by lentiviral transduction (Figures 2a,b). Upon treatment with 5 μM doxycycline, we followed the expression of gRNA-2 and the scramble control over multiple timepoints (Figure 2c). The gRNA levels peaked at day three, which corresponded to the greatest decrease (more than two-fold) in *c-MYC ANCOR* expression (Figure 2d).

To define the impact on DNA replication at the *c-MYC* origin, we quantified the nascent DNA (nasDNA) abundance within the origin and found a stark decrease in nasDNA generated near the origin (between 2.4 – 4.5 fold), (Figure 2e). Importantly, no substantial changes in cell growth were observed as no differences in MYC protein levels between the control and gRNA-2 expressing cells were observed upon doxycycline treatment and after its suspension (Supplementary Figure 2e). These findings suggest that the effect on nasDNA was independent of *c-MYC* levels ^32,33^.

To complement the loss-of-function experiments and to ensure the consistency of the observed phenotype, gain-of-function experiments were performed using the CRISPR activator (CRISPRa, CRISPR-VP64) system ^34^. To that end, gRNA-2 was delivered in stably expressing dCas9-VP64 HEK 293 cells, wherein *c-MYC* is not actively expressed in contrast to K562 cells ^35,36^. This led to robust upregulation of *c-MYC ANCORs* lasting up to five weeks, paralleled by an increase in *c-MYC* expression (Figure 2f). nasDNA abundance was measured in dCas9-VP64 HEK 293 cells stably expressing gRNA-2 or the scramble control. We observed enrichment of newly synthesized DNA strands at the origin of replication 127 bp upstream of the *c-MYC* TSS upon expression of *c-MYC ANCORs* as compared to controls, hence supporting the hypothesis that *c-MYC ANCORs* could promote or aid licensing and firing of the *c-MYC* replication origin (Figure 2g). Interestingly, we also observed an increase in cell growth likely due to the increased levels of *c-MYC* protein (Supplementary Figure 2f).

Collectively, these results delineate the role of *c-MYC ANCORs* in the formation of the *c-MYC* replication origin and underscore how alterations in their levels may perturb the integrity of the replication process at the targeted locus.

### ORC1 engagement of H2A.Z by G1/S phase transcribed RNAs controls DNA replication.

**Figure 3|:**
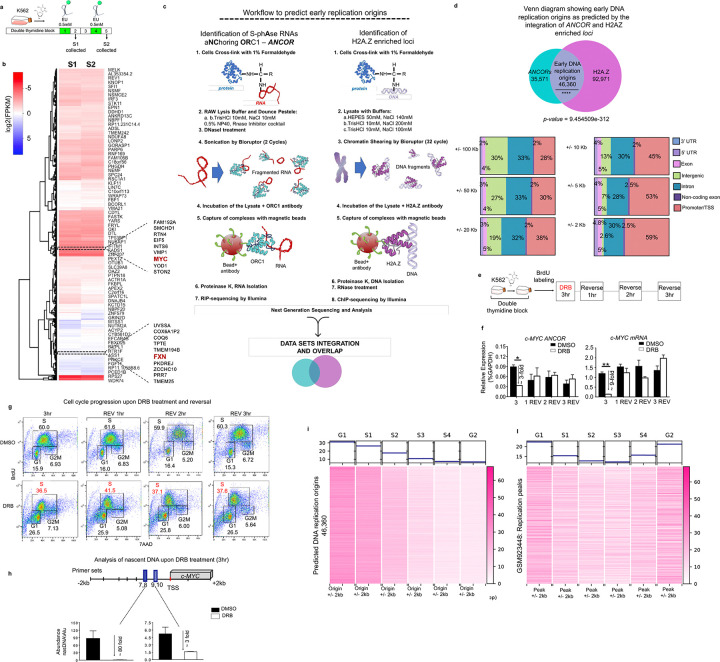
ORC1 engagement of H2A.Z by G1/S phase RNAs mark early replication origins. **a**. Outline of the experimental setup used to identify nascent RNAs. Upon synchronization in the G1/S phase by double thymidine block, the growth medium was supplemented with the EU RNA analog at the indicated time points: S1 (2 hr) and S2 (5 hr). EU-labeled nascent RNA was collected at S1 and S2 by Click-iT conversion for RNA sequencing. **b**. Heatmap showing gene expression profiles of K562 at S1 (2 hr) and S2 (5 hr). Welch’s t-test: p-value = 0.02993 **c**. Workflow employed to predict early replication origins through the integration of S-ph**A**se RNAs a**NC**horing **OR**C1 (***ANCOR***), detected by RNA immunoprecipitation with anti-ORC1 antibody followed by sequencing (RIP-seq) and H2A.Z-enriched sites captured by Chromatin Immunoprecipitation sequencing (ChIP-seq); **d**. Upper panel, Venn diagram showing early DNA replication origins as predicted by the integration of *ANCOR* and H2AZ-enriched *loci*. Lower panel, annotation of the predicted early DNA replication origins located within +/−100kb, +/−50kb, +/−20kb, +/−10kb +/−5kb and +/−2kb of the transcription start site (TSS). A significant proportion of the predicted early DNA replication origins are within close proximity to promoters and/or TSS of protein coding genes; **e**. Schematic showing K562 synchronization in G1/S phase cycle followed by incorporation of BrdU into nasDNA and pharmacological inhibition of transcription using DRB (100 mM). Samples were collected 3 hr after DRB treatment and every hour upon DRB reversal over the following 3 hr; **f**. Reversible downregulation of *c-MYC ANCOR* and *c-MYC mRNA* expression by qRT-PCR upon DRB treatment and the reversal. The mean ± S.D. was calculated for three independent experiments (n=3). Student’s t-test, ***: p value 0.0006 (*c-MYC mRNA*) and **: p value 0.0223 (*c-MYC ANCOR*); **g**. Flow cytometry analysis showing nonreversible inhibition of nas-DNA synthesis at all selected time points; **h**. DNA abundance within the *c-MYC locus* significantly decreases upon DRB treatment only within the region encompassing the origin (7–8 and 9–10). **i**. Heatmap showing analysis of the K562 Repli-Seq signal in G1/G1b, S1, S2, S3, S4, and G2 cell cycle fractions (GSE34399) centered at the ANCOR-H2A.Z predicted 46,360 early DNA replication origins in K562; **l**. Heatmap showing analysis of Repli-Seq signal centered at the replication peaks (GSE34399) in K562 cells.

The identification of *c-MYC ANCORs* and the RNA binding features retained by ORC1 and H2A.Z suggested the involvement of RNA in the formation of replication origins and their chromatin status at the *c-MYC* locus. Therefore, we sought to explore the extent of ORC1–S-phase RNA association in other genomic loci with respect to the presence of DNA replication origins. To this end, we assessed the transcriptional profile of G1/S phase transcribed RNAs upon cell synchronization by performing nascent RNA sequencing (nasRNA-seq) at earlier (S1) and later (S2) timepoints after the release in the S-phase over the course of 5 hr ^23^, (Figure 3a). This approach led to the identification of 10,087 genes expressed in the earlier S1 timepoint and 10,171 in the later S2 timepoint. Both included *c-MYC* within the group of genes displaying the highest expression (Figure 3b, Supplementary Figure 3a) and *FXN* among the group of genes displaying the lowest expression (Figure 3b, Supplementary Figure 3a). *FXN* is harbored in a mid-late replicating *locus*, and its replication profile has been well characterized owing to its involvement in Friedreich’s Ataxia ^9,37,38^. Next, we examined the interaction between ORC1 and the RNAs transcribed in the first 3 hr of S phase to determine whether we could establish a correlation between the presence ORC1-RNA binding and early replication origin formation. We uncovered 81,931 predicted loci associated with *ANCORs*, thereby expanding on the potential global nature of transcripts showing features similar to those of *c-MYC ANCORs*. Since Long *et al.* have proposed a function for H2A.Z in licensing DNA replication loci, we investigated the linkage between the genomic location of *ANCORs* and enrichment of H2A.Z in K562 cells (Figures 3c). Over 56% of loci associated with *ANCORs* overlapped with H2A.Z-enriched genomic locations, with 46,360 (*ANCORs*/H2A.Z) predicted early replication origins (Figures 3c,d). The *ANCOR*-defined origins were broadly distributed across the genome including promoters/ transcription start site (TSS), gene bodies (3’ UTR, 5’ UTR, exons, and introns) and intergenic regions. Specifically, when narrowing the window for origin localization from +/−100kb to +/−0.5kb with respect to TSS, the number of predicted early DNA replication origins only decreased by two-fold, from 43,188 to 19,725. A significant proportion of these loci were located within the proximity of promoters and/or TSS of protein-coding genes, specifically 58% within 2kb or 67% within 0.5kb as compared to 28% within 100kb (Figure 3d, Supplementary Figure 3b,c). Overall, these results confirm that most DNA replication origins tend to localize in closer proximity to the promoters and/or TSS of protein-coding genes ^2,39^.

To verify whether pharmacological inhibition of transcription could lead to alterations in S phase cell cycle progression, K562 cells were synchronized by double thymidine block ^36^ and treated with the RNA Polymerase II (RNAPII) inhibitor 5,6-Dichlorobenzimidazole-1-β-D-ribofuranoside (DRB) upon release into S phase. Concurrently, 3.5 μm 5-bromo-2′-deoxyruridine (BrdU) was added to the cells to monitor the nascent DNA synthesis by flow cytometry following transcriptional inhibition. We took advantage of the reversibility features of DRB and examined the effect of global transcriptional inhibition 3 hr upon DRB treatment and every hour for 3 hr after DRB reversal (treatment outline shown in Figures 3e–g). We monitored transcriptional inhibition by measuring *c-MYC ANCOR* levels by qRT-PCR (Figure 3f). Strong downregulation of *c-MYC ANCOR* and *c-MYC* mRNA (nearly 3- and 9-fold change, respectively) was noted 3 hr after DRB treatment. However, these changes were transient and reversible, and the expression returned to baseline during DRB reversal (Figure 3f). By contrast, non-reversible inhibition of cell cycle progression was observed at all selected time points upon DRB reversal, as evidenced by the sharp reduction to 38% of the S phase cell population in DRB-treated cells as compared to the 60% of the S phase population in DMSO-treated control cells (Figure 3g). Consistently, this pattern was associated with a decrease in nasDNA synthesis at the *c-MYC locus* and loss of ORC1 and H2A.Z enrichment at the *c-MYC* origin upon transcriptional inhibition (Figure 3h, Supplementary Figure 3d). Similarly, DRB treatment of another cell line, HL60, resulted in impairment of DNA synthesis, followed by a drastic reduction in the S phase cell population to nearly 30% in DRB-treated cells as compared to 50% in the mock control cells (Supplementary Figure 3e).

Lastly, using a publicly available data set (GSE34399) of nascent DNA sequencing (Repli-Seq), obtained from K562 sorted at different cell cycle stages (G1/G1b, S1, S2, S3, S4, G2), we interrogated the distribution of early replication origins associated with *ANCORs* and enriched in H2.AZ against all the replication origin captured by Repli-Seq (Figure 3i). Briefly, the Repli-Seq dataset was intersected with our dataset of origins initiated by *ANCORs* (Figures 3d,i). Stronger signal intensity was detected in the G1 and S1/S2 cell fractions when the analysis was computed with respect to the *ANCOR* predicted 46,360 DNA replication origins, while it gradually declined in the later phases (Figure 3i). In contrast, the signal appeared randomly distributed across the different cell-fractions when the signal was not centered across the *ANCOR* predicted replication origins but around the replication peaks ^40^(Figure 3l). These results are consistent with the hypothesis that *ANCOR* enriched loci mark early DNA replication origins.

Taken together, these data demonstrate a global interaction of RNAs transcribed in G1/S phase and ORC1, in association with H2A.Z that provides a tool for the detection of early replication origins across the genome. On a related note, these findings support the hypothesis that *ANCOR*s are involved in the initiation of DNA replication.

### ORC1 engagement and H2A.Z enrichment is lost upon inhibition of G1/S phase transcribed RNAs.

**Figure 4|:**
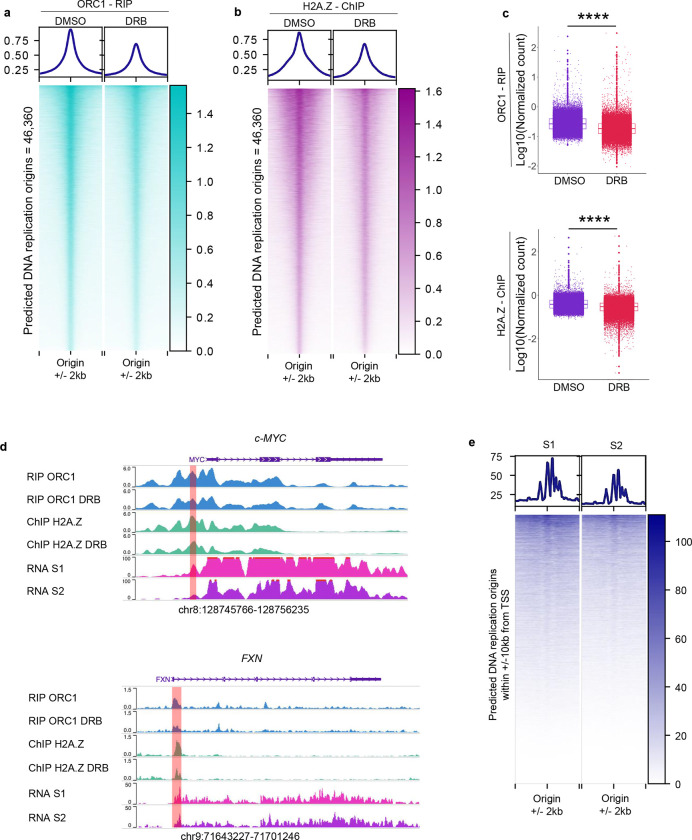
Impaired ORC1 engagement and loss of H2A.Z upon inhibition of *ANCORs*. **a**. Heatmap showing a decrease in ORC1 enrichment by RIP-Seq at 46,360 predicted early DNA replication origins upon DRB treatment as compared to DMSO mock control in K562 cells. Welch’s t-test: *p-value* < 2.2e-16; **b**. Heatmap showing a decrease in H2A.Z enrichment by ChIP-Seq at 46,360 early DNA replication origins in response to DRB treatment as compared to DMSO mock control in K562 cells. Welch’s t-test: *p-value* = 4.473e-14; **c**. Box plot showing a global reduction in ORC1-RIP (upper panel) and H2A.Z-ChIP signals upon treatment with DRB in K562; **d**. Genomic snapshots of the *c-MYC* and *FXN loci* depicting the impact of pharmacological inhibition of transcription using DRB on ORC1 and H2A.Z enrichment at the respective origin sites. Transcriptional profile of the *c-MYC* and *FXN loci* for the S1 and S2 time points are presented. The rose gold bar indicates the well-documented *c-MYC* origin [4, 6], and the predicted origin by the *ANCORs* association for *FXN*; **e**. Heatmap showing the transcriptional profiles of K562 at S1 and S2 centered on *ANCOR*-predicted replication origins within +/−10kb of the transcription start site (TSS). The higher transcriptional activity in S1 supports the association of *ANCOR*-associated *loci* with early DNA replication origins.

The changes in cell cycle progression upon DRB treatment prompted us to compare ORC1 engagement with respect to H2A.Z distribution across the 46,360 predicted early DNA replication origins (Figure 3c,d). We performed these experiments in synchronized cells and applied pharmacological inhibition of transcription by DRB in the first 3 hr after release from G1/S. Blocking early G1/S-phase RNAs resulted in a significant reduction in ORC1-RNA interactions as compared to the DMSO control (*p-value* < 2.2e^−16^) and corresponded to depletion of H2A.Z (*p-value* = 4.473e^−14^) at the respective loci (Figures 4a–c). Importantly, when inspecting the *c-MYC* locus, we confirmed that DRB-mediated inhibition of transcription resulted in depletion of ORC1-RNA interactions and H2A.Z enrichment at the *c-MYC* origin (Figure 4d). This also held true for the *FXN locus* that clustered with the lowest expressing group (Figure 3b). Indeed, the expected decrease in ORC1-RNA interactions associated with reduced enrichment of H2A.Z at the *FXN* origin upon inhibition of transcription (Figure 4c). Next, we stratified the K562 transcriptional profiles at the earlier (S1) and later (S2) timepoints after the release in S phase centering the analysis on the *ANCOR*-predicted replication origins over a distance of +/−10kb from the TSS. Consistently, we detected higher transcriptional activity in the S1 timepoint as compared to the S2 timepoint (*p-value* = 0.02993), corroborating the hypothesis that *ANCOR*-associated loci mark early replication origins (Figure 4e).

These findings demonstrate that inhibition of transcription during the early S phase impairs both ORC1 engagement and H2A.Z enrichment, pointing to an RNA-mediated mechanism coordinating the formation of early replication origins.

### Disruption of *ANCORs* perturb DNA replication dynamics.

**Figure 5|:**
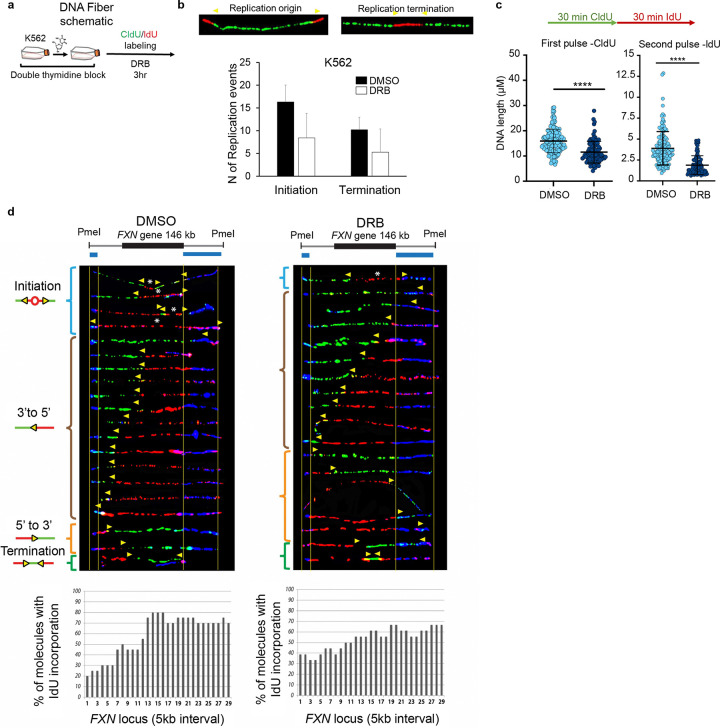
DNA Replication dynamics is perturbed by inhibition of ANCOR levels. **a**. Depicted the experimental design for DNA fiber analysis. K562 cells synchronized at G1/S phase by double thymidine block were treated with DRB for 3hrs upon release into the S phase, and sequential additions of 5-iodo-2′-deoxyuridine (IdU) and 5-chloro-2′-deoxyuridine (CldU) were used to follow the progression of replication forks during transcriptional inhibition; **b**. Quantification of DNA fibers at the “origin initiation” and “origin termination” in K562 DRB-treated cells as compared to DMSO; **c**. Quantification of DNA fibers during the two consecutive pulses with IdU and CldU, respectively for K562 DRB-treated cells compared to DMSO; **d**. Single molecule analysis of replicated DNA (SMARD) performed on *FXN locus*. Origin initiation, DNA replication fork progression (3’ to 5’ and 5’ to 3’), and origin termination were monitored upon DRB treatment and DMSO control. Percentage of molecules with IdU incorporation is indicated in the column chart below each respective condition.

We sought to determine whether disruption of *ANCOR’s* transcription in G1/S phase, would destabilize the origin formation and the replication process. To this end, we monitored the replication fork dynamics genome-wide by leveraging DNA fiber analysis at single-molecule resolution ^41,42^ (Figure 5a). Synchronized K562 cells were treated with DRB for 3 hr upon release into S phase, while sequential additions of 5-iodo-2′-deoxyuridine (IdU) and 5-chloro-2′-deoxyuridine (CldU) ^42^ were included in the medium to follow the progression of the replication forks during transcriptional inhibition (Figure 5a). Upon *ANCOR* downregulation, nasDNA production was impaired and both DNA replication initiation and fork progressions were decreased in DRB-treated K562 cells as compared to DMSO-treated cells (Figures 5b, c).

To elucidate the impact of *ANCOR* transcriptional inhibition on DNA replication progression within a specific gene locus, we performed single molecule analysis of replicated DNA (SMARD) on *FXN*, a locus amenable to this approach, unlike *c-MYC* (Figures 3b, 4d) ^9,43^. Strikingly, DNA replication fork progression at the *FXN* locus was disrupted by DRB-mediated transcription inhibition. The abnormal replication pattern exhibited a reduced number of initiation events, altered replication progression from 5’ to 3’, and increased terminating events when compared to the DMSO control (Figure 5d), thus confirming that *ANCORs* play a critical a role in maintaining the integrity of the replication process.

Our findings reveal that downregulation of *ANCORs* halts the DNA replication program (Figures 3e–g, Supplementary Figure 3e), ultimately leading to a broad and deleterious perturbation of DNA replication dynamics.

In conclusion, these results show that enforced expression of *ANCORs* promotes the initiation of DNA replication origins, providing a potential strategy to modulate the origins of replication at both local and global levels in mammalian cells.

## DISCUSSION

DNA replication initiation needs to be molecularly and temporally coordinated to ensure efficient DNA synthesis and to preserve cell-specific replication dynamics. Our investigation examined the molecular steps leading to the “*initiation*” of the DNA replication. Specifically, we focused on the unexplored contribution of RNAs transcribed in the G1/early S phase of the cell cycle as the masterminds coordinating the engagement of ORC1 (the first subunit of the pre-replication complex) with H2A.Z, enabling DNA replication in a timely and selective fashion.

Through the identification of ***ANCOR*s**, we delve into the role of RNA in maintaining the integrity of DNA replication origin activity and provide evidence of the mechanism governing the *initial events* of DNA replication. We demonstrate that changes in the expression of *ANCORs* result in perturbation of the replication profile. Additionally, we describe a tool to regulate site-specific gene replication in human cells by means of RNA.

By linking the first subunit of the origin recognition complex ORC1 and the histone variant H2A.Z, *ANCORs* showcase unexpected abilities to control gene replication at precise genomic sites for future translational applications. Indeed, control of gene replication “*in loco*” holds promise for precise intervention in diseases associated with dysfunctional DNA replication. The lack of understanding how DNA replication is initiated at specific sites has represented a major roadblock to the development of successful therapeutics for these diseases. Herein, we present evidence to harness RNA molecules as a tool to control locus-specific replication in human cells.

GC-rich elements have been identified as integral components of replication origins in human cells. These GC-rich sequences serve as binding sites for key replication factors, including the origin recognition complex (ORC) ^27^. In unraveling the mechanisms governing mammalian DNA replication, we have identified a distinctive RNA sequence (RM9) embedded within the *c-MYC* origin (region 9–10) that can bind ORC1 (Figures 1e–g). With unrelated and GC-poor RNA (*i.e.* MYC1–3) or homologous DNA (dRM9) sequences, only a weak or no interaction was detected, respectively, indicating that ORC1-RNA complex formation has both sequence and structural requirements.

By examining a range of ORC1 conformations *in silico*, mainly to vary the orientation between the N- and C- terminals, we modeled ORC1-RNA recognition. The ORC1 N- and C-terminals include the BAH domain (1–200aa) and AAA+ domain (201–782aa, ATPases associated with diverse cellular activities)/winged-helix domain (WHD, 783–861aa), respectively. At present, the complete architecture of the ORC1 subunit structure remains poorly characterized owing to the presence of flexible regions, referred as disordered domains, that connect the N- and the C-terminal regions. These disordered domains within ORC1 as well as the conformational dynamics of its AAA+ domain have been considered functional to its role in inducing the replication machinery ^13^. Our theoretical model demonstrates that when the BAH domain is part of the binding region, dRM9 lies in close proximity to ORC1 during the ORC1b-dRM9 and ORC1c-dRM9 simulations. When the BAH domain is not included in the binding site in the ORC1a state, dRM9 deviates from its initial position. By contrast, RM9 loses its fold when protein complex formation involves the BAH domain and efficient targeting when the BAH domain is displaced away from the interacting region.

Taken together, these data suggest that the BAH domain is not involved in complex formation with RNA and provide a model wherein the BAH domain needs to be structurally free to interact with the histones. In this proposed model, the ORC1a conformation exposes a specific binding site that perfectly fits RM9 but cannot accommodate dRM9. Accordingly, RNA would promote a long-range effect on the ORC1 dynamic by affecting the conformational equilibrium of the protein and targeting the ORC1a conformation in a pocket formed by ORC1 disordered and AAA+ domains. This change in the conformational equilibrium would then facilitate the engagement of the BAH domain for subsequent chromatin binding.

Pharmacological inhibition of transcription by DRB treatment yielded compelling evidence of a significant reduction in ORC1-RNA interactions and H2A.Z enrichment globally, consistent with the decrease in DNA replication initiation and fork progression detected by fiber analysis. Compared with control treatment, DRB treatment also disrupted DNA replication fork progression (reduced initiating events, altered replication progression from 5’ to 3’, and increased terminating events) at the *FXN locus* as captured by SMARD analysis in K562 DRB-treated cells as compared to the control (Figures 4–5). These results shed light upon the interplay between G1/S phase transcription, chromatin dynamics, and establishment of DNA replication origins, supporting the role of RNA in the formation of active replication origins.

Mas *et al.* (2023) have reported an interaction between RNAs transcribed at active origins and ORC1. They linked this interaction to ORC1’s dynamic association with chromatin and concluded that disruption of ORC1-RNA binding *“has an in impact on replication origin activity*” ^44^. However, there are substantial differences between our study and the one by Mas *et al.* First and foremost, the authors suggest a mechanism by which “*RNA binding favors ORC1 chromatin release*” ^44^. By contrast, we identify *ANCORs* as the recruiters of ORC1 and initiators of DNA replication origins in human cells. We delineate the RNA-mediated molecular events responsible for the initiation of early replication origins. Additionally, we reveal a longstanding question on how distribution and activation of replication origins are controlled in mammalian cells. We also show that replication can be initiated in a *locus*specific manner by induction of *ad hoc* RNA (*ANCORs*), with *ANCOR*-like transcripts reverting genetic abnormalities caused by dysfunctional DNA replication. Conversely, Mas *et al.* describe RNAs as behaving as a “*facilitator*” for the release of ORC1 from the chromatin. Therefore, replication cannot be initiated by the same RNA in their model. We instead propose that *ANCORs* act as a “*conductor*” of the DNA replication process and can thus initiate replication. In simple terms, we posit that *ANCORs* control ORC1 engagement of H2A.Z and firing of early DNA replication origins, thus serving as the leading players in the formation of replication origins. Nevertheless, the stated RNAs could subsequently favor ORC1 release from the chromatin as described ^44^.

**Figure 6|:**
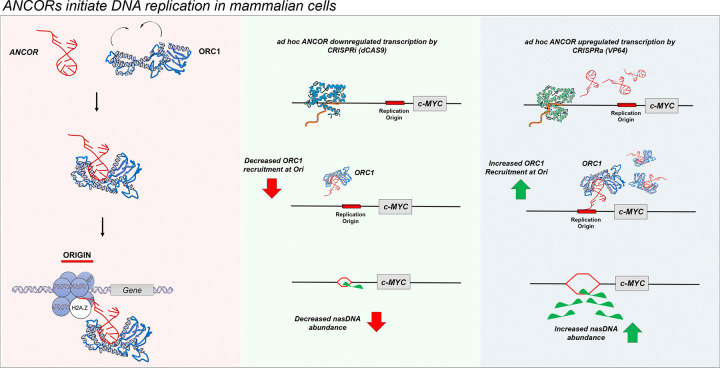
Proposed model for DNA origin formation. Depicted a schematic of ORC1 conformational changes that enable binding to RNA and formation of the RNP-complex. The RNA engagement of ORC1 to the origin site through H2A.Z enables licensing of early DNA replication origins (**left box**). Disruption of *c-MYC ANCOR* transcription by CRISPRi (dCAS9), affects ORC1 engagement at replication origin decreasing nasDNA synthesis (**middle box**). By contrast, induced *c-MYC ANCOR* transcription by CRISPRa (VP64), increases ORC1 engagement at replication origin increasing nasDNA synthesis (**right box**)

The molecular steps involved in the formation of DNA origins, as well as its modulation, support a model wherein *ANCORs* coordinate the engagement of ORC1 with H2A.Z across the genome, thereby licensing the initiation of early replication origins (Figure 6). Importantly, our model has a significant therapeutic implication, i.e., an RNA-guided strategy to control DNA replication. Indeed, *ANCOR*-specific downregulation by CRISPRi severely impacted the activity of the *c-MYC* origin activity, whereas *locus*-specific upregulation of *ANCOR* by CRISPRa led to increased production of nasDNA, a schematic of the proposed model is depicted in Figure 6.

In conclusion, our study identifies *ANCOR*s as the pacesetters for the formation of early DNA replication origins and alludes to the broader involvement of *ANCOR*s as *initiators* of DNA replication origins. Furthermore, the data reveal an RNA-centered mechanism to control and modulate DNA replication in eukaryotic cells at specific sites, thereby providing a novel tool to treat diseases caused by DNA replication abnormalities (Figure 6).

## MATERIALS AND METHODS

### Cell culture

The chronic myeloid leukemia K562 and acute myeloid leukemia HL-60 cell lines were procured from ATCC and cultured in RPMI-1640 media (Corning, Cat. No. 10–040-CM) supplemented with 10% fetal bovine serum (FBS) without antibiotics at 37°C in a humidified atmosphere with 5% CO_2_. HEK 293 cells obtained from ATCC were cultured in DMEM (Corning, Cat. No. 10–013-CV) with 10% FBS without antibiotics at 37°C in a humidified atmosphere with 5% CO_2_.

### RNA and DNA isolation

**Total RNA** isolation was conducted following a previously described protocol ^45^. Subsequently, all RNA samples utilized in this study were subjected to DNase I treatment (10 U of DNase I per 3 mg of total RNA) at 37°C for 1 hr in the presence of RNase inhibitor to remove any contaminating DNA. To ensure complete DNA removal, the RNA samples were further purified using acidic phenol (pH 4.3). For cDNA synthesis, either Random Primers (Invitrogen) or gene-specific primers were employed in conjunction with Reverse Transcriptase (Roche Applied Science), following the manufacturer’s guidelines. The resulting cDNA was purified using the High Pure PCR Product Purification Kit (Roche Applied Science) ^45^.

**Nuclear RNA** was isolated using a modified method based on nuclei isolation protocols ^46^. Approximately 50 million viable cells were washed with ice-cold PBS containing 5 mM vanadyl complex and 1 mM PMSF. The cells were then resuspended in ice-cold lysis buffer composed of 1x Buffer A (10 mM HEPES-NaOH pH 7.6, 25 mM KCl, 0.15 mM spermine, 0.5 mM spermidine, 1 mM EDTA, 2 mM Na butyrate), 1.25 M sucrose, 10% glycerol, 5 mg/mL BSA, 0.5% NP-40, and supplemented with various protease inhibitors, vanadyl complex, and RNase inhibitor (RNAguard; Amersham Biosciences). The samples were incubated at 0°C for approximately 10 min and homogenized through multiple strokes in a Dounce homogenizer. The pelleted nuclei were further processed and centrifuged, and the resulting nuclear RNAs were extracted. All RNA samples, including total, cytoplasmic, and nuclear RNA, were treated with DNase I to remove any contaminating DNA and then extracted with acidic phenol (pH 4.3). Polyadenylated and non-polyadenylated RNA fractions were separated using the MicroPoly(A)PuristTM purification kit (Ambion). Subsequently, cDNA synthesis was performed with Random Primers and Transcriptor Reverse Transcriptase following the manufacturer’s recommendations. The cDNA was purified using a High Pure PCR Product Purification Kit.

**Genomic DNA** isolation was performed following a previously described method ^24^. Briefly, cell pellets were resuspended in lysis buffer containing 0.5% SDS, 25 mM EDTA (pH 8), 10 mM TRIS (pH 8), and 200 mM NaCl. After treatment with RNase A (Epicentre) at 37°C for 20 min and Proteinase K (Roche) overnight at 65°C, high-quality genomic DNA was extracted using PhenolChloroform (Sigma, pH 8) and precipitated overnight with isopropanol. The genomic DNA was then resuspended in TE buffer (pH 8) and stored at 4°C.

**Nascent RNA/DNA capture** was performed using a Click-iT^®^ Nascent RNA Capture Kit (Ther-moFisher) according to the manufacturer’s instructions with minor modifications. Briefly, 1. **Labeling the cells with 5-ethynyl uridine (EU)/Ethynyl 2-deoxyuridine (EdU)**. 200 mM EU or 30 mM EdU stock solutions were added to the cells to a final concentration 0.5 mM or 30 μM, respectively. 2. **Incubation**. The cells were incubated for 1 or 2 hr. 3. **RNA/DNA isolation**. The cells were harvested, and the RNA/DNA were isolated and dissolved in 14 μL of H_2_O. 4. **Biotinylation of RNA/DNA by Click reaction**. Click-iT^®^ reaction cocktail (50 μL per reaction) was prepared according to the manufacturer’s instructions. A mixture containing 1x Click-iT EU buffer; 2 mM CuSO_4_; 1 mM Biotin azide; 13.25 μL of the isolated RNA; 10 mM Click-iT^®^ reaction buffer additive 1; and 12 mM Click-iT^®^ reaction buffer additive 2 was prepared. After adding each component, the reaction cocktail was gently mixed. The addition of the Click-iT^®^ reaction buffer additive 1 stock initiated the click reaction between the EU-RNA/EdU-DNA and biotin azide. Subsequently, the Click-iT^®^ reaction buffer additive 2 was added and incubated for 30 min with gentle vortexing. 5. **RNA/DNA precipitation**. 1 μL of UltraPure^™^ Glycogen, 55 μL of 7 M ammonium acetate, and 750 μL of chilled 100% ethanol were added to the click reaction and incubated at −70°C for at least 30 min. After centrifugation, the pellet was dissolved in 125 μL of H_2_O. 6. **Binding biotinylated RNA/DNA to Dynabeads**^**®**^ **MyOne**^**™**^ **Streptavidin T1 magnetic beads (ThermoFisher)**. The RNA/DNA binding reaction mixture included: 125 μL 2xClick-iT^®^ RNA binding buffer; 2 μL Ribonuclease Inhibitor or 2 μL of water for DNA; and 125 μL of the isolated biotinylated RNA/DNA. The RNA binding reaction mixture was heated at 68–70°C for 5 min, and 50 μL of bead suspension was added into the heated RNA binding reaction mixture. The tube containing the RNA/DNA binding reaction was incubated at room temperature for 30 min while gently vortexed to prevent the beads from settling. The beads were immobilized using a magnet and washed 5 times with 500 μL of Click-iT^®^ reaction wash buffer 1 and 5 times with 500 μL of ClickiT^®^ reaction wash buffer 2. Finally, the beads were resuspended in 50 μL of Click-iT^®^ reaction wash buffer 2, and the captured RNA was immediately reverse transcribed to cDNA. The captured DNA was released into 50 μL of boiling water and used in qPCR analyses.

**List of primers** used to determine nasDNA abundance on the *c-MYC* locus:

**Table:** 

Forward myc3	5’-TCAGAAAAAATTGTGAGTCAGTGA-3’
Reverse myc4	5’-TTGTGGACCGAGCCGGGGGAGTCA-3’
Forward myc7	5’-ACAGGCAGACACATCTCAGGGCTA-3’
Reverse myc8	5’-ATAGGGAGGAATGATAGAGGCATA-3’
Forward myc9	5’-CTACACTAACATCCCACGCTCTGA-3’
Reverse myc10	5’-AACCGCATCCTTGTCCTGTGAGTA-3’
Forward myc15	5’-GAACGGAGGGAGGGATCGCGCTGA-3’
Reverse myc16	5’-GTGCAAAGTGCCCGCCCGCTGCTA-3’
Alu primer set (Alu115); Forward	5’-CCTGAGGTCAGGAGTTCGAG-3’
Alu primer set (Alu115); Reverse	5’-CCCGAGTAGCTGGGATTACA-3’

### qRT-PCR

**Sybr Green analysis**: iQ Sybr Green supermix (Biorad, Hercules, CA) was employed with the following thermal cycling parameters: an initial denaturation step at 95°C for 10 min, followed by 40 cycles of denaturation at 95°C for 15 seconds, annealing at 60°C for 1 min, and extension at 72°C for 1 min. **TaqMan analysis**: Hotstart Probe One-step qRT-PCR master mix (USB) was used with the following temperature profile: an initial step at 50°C for 10 min, a denaturation step at 95°C for 2 min, followed by 40 cycles of denaturation at 95°C for 15 seconds and annealing/extension at 60°C for 60 seconds.

#### Primers and TaqMan probes used in real time PCR.

Eukaryotic 18S rRNA Endogenous Control: ABI Cat. # 4310893E, Human GAPDH Endogenous Control: ABI Cat. # 4310884E, Human MYC mRNA: ABI Cat. # Hs00153408_m1, c-MYC ANCOR custom design (FRW-Primer: TCTGGGTG-GAAGGTATCCAA, TaqMan probe: CCAACAAATGCAATGGGAGT, REW-Primer: TTGGA-GAGCGCGTTATGAAT).

#### Primers used for strand-specific real-time RT PCR (Sybr Green).

Reverse Transcriptase primer for *c-MYC ANCOR*: 5’- AAC CGC ATC CTT GTC CTG TGA GTA -3’; qRT-PCR primers: Forward: 5’- ACA GGC AGA CAC ATC TCA GGG CTA -3’, Reverse: 5’- ATA GGG AGG AAT GAT AGA GGC ATA -3’.

### Antibodies

Rabbit polyclonal anti-ORC1 (Abcam Catalog No. ab85830); goat anti-rabbit (Invitrogen, Catalog No. 32460); rabbit polyclonal human Histone H2A.Z – ChIP Grade (Abcam Catalog No. ab4174); mouse monoclonal beta Actin (SantaCruz, sc-81178); goat anti-mouse (Invitrogen 32430); rabbit monoclonal c-MYC (Cell Signaling Technology, D84C12).

### Recombinant proteins

Recombinant human origin recognition complex subunit 1 (ORC1) (Cusabio Catalog N. CSB-BP621667HU(A4)); mononucleosome H2A.Z (Active Motif, Cat. No. 81072)

### RNA pull-down assay (RNAP)

The RNA probes were custom-designed to investigate ORC1 binding to RNA structures transcribed within the c-MYC locus, including the c-MYC promoter, c-MYC gene and c-MYC origin. The specific set of synthetic biotinylated RNA oligonucleotides used in the RNAP is listed as follows:

**Table:** 

RM9	5’-GAGAACGCACUGCGCGCCCACCGCCACGCCACGCGCGUAC-3’ (BTN)
MYC1	5’-CAGCCGCCCACUUUUGACAGGCCUGGGCGGGCUUCGCUUA-3’ (BTN)
MYC2	5’-GAGCUCCCAAAUCUCUCCAGAUCUGCUAUCUCUCCUUCCU-3’ (BTN)
MYC3	5’-GAUGGGAGGAAACGCUAAAGCCCAAGGUUUCAGAGGUGAU-3’ (BTN)

RNAP was conducted in triplicate following a modified protocol adapted from Pierce^™^ Magnetic RNA-Protein Pull-Down Kit (Thermo Scientific^™^, No.: 20164). Briefly, 3 μg of biotinylated RNA was denatured at 90°C for 2 min and then allowed to fold into proper RNA structures at 25°C for 20 min. 3×10^6^ cells were resuspended in 1 ml of RIP Buffer containing 150 mM KCl, 25 mM Tris pH 7.4, 0.5 mM DTT, 0.5% NP40, 1 mM PMSF, and protease inhibitor (Roche Complete Protease Inhibitor Cocktail Tablets). Cells were homogenized with a homogenizer for 20 strokes, and debris was pelleted and discarded by centrifugation at 13,000 rpm for 10 min. 0.1 μg of biotinylated RNA was incubated with 1 mg of cell lysate at room temperature for 1 hr. 60 μl of streptavidin agarose beads (Invitrogen) were added to the mix and incubated at room temperature on a shaker. The beads were then washed 10 times with RIPA buffer and boiled at 100°C in 30 μl of SDS sample buffer. Finally, the beads were removed using a magnetic field, and the samples were analyzed by western blot.

### Cell synchronization and drug treatment

To induce an early S phase block of the cell cycle, the Double Thymidine block method was employed as previously described ^36^. Briefly, K562 and HL-60 cells were grown overnight to 70–80% confluence, washed twice with 1x PBS, and then cultured in DMEM (10% FBS) supplemented with 2.5 mM thymidine for 18 hr (first block). Subsequently, thymidine was washed out with 1xPBS, and the cells were cultured in DMEM (10% FBS). After 8 hr, the cells were treated with thymidine again for 18 hr (second block). Cells were then released from the block as previously described ^36^.

K562’s cell cycle synchronization in G phase was achieved by incubating the cells for 24 hr with Nocodazole (15mM) as previously described ^47^.

Synchrony was assessed by flow cytometry analysis after propidium iodide 1mg/mL (Sigma)- or 7AAD vs BrdU cell staining, using a LSRII flow cytometer (BD Biosciences) at the Harvard Stem Cell Institute/Beth Israel Deaconess Medical Center flow cytometry facility.

**DRB treatments** were carried out as previously described ^24^. Briefly, after release from double thymidine block, cells were treated with 100 μM of DRB (Sigma Aldrich).

**EU (5-ethynyl uridine, to label nasRNA) and or EdU (Ethynyl 2-deoxyuridine to label nasDNA)** were purchased from Click Chemistry Tool (CCT) and added to cell cultures at a final concentration of 0.5mM (EU) or 30μM (EdU). Cells were cultured for 1 hr with EU and 40 min with EdU to achieve maximum labeling of nasRNA and nasDNA.

### BrdU staining and flow cytometric analysis of the S-phase cycle

Minor modifications were made to the manufacturer’s instructions provided in a BD Pharmingen BrdU Flow Kit (Catalog No. 559619). Briefly, K562 cells were cultured in cell culture medium and incubated with BrdU at a final concentration of 10 μM for a pulsing period of 45 min. After the pulsing period, cells were resuspended in 100 μL of BD Cytofix/Cytoperm Buffer and washed with 1 mL of 1X BD Perm/Wash Buffer. The cells were then incubated with BD Cytoperm Permeabilization Buffer at all steps, keeping the samples on ice to facilitate fixation and permeabilization of the cell membranes. Following permeabilization, the cells were resuspended in 100 μL of diluted DNase, which was prepared at a concentration of 300 μg/mL in DPBS. This resulted in a dilution of 30 μg of DNase per 106 cells. The cells were incubated with DNase for 1 hr at 37°C. After the incubation, a wash step with 1 mL of 1X BD Perm/Wash Buffer was performed. To detect BrdU and total DNA content, the cells were stained with a Fluorochrome-conjugated anti-BrdU Antibody and 7-aminoactinomycin D (7-AAD) fluorescent dye. The staining was carried out for 20 min at room temperature The stained cells were then analyzed using a flow cytometer to determine the incorporation of BrdU and DNA content. Cell acquisition and analysis were performed on BD LSRFortessa (BD Biosciences, Franklin Lakes, NJ, USA) using BD FACSDivaTM software (BD Bioscience, Franklin Lakes, NJ, USA). Analysis was performed using Flowjo software (Flowjo LLC, Ashland, OR, USA).

### Western Blotting analysis

#### ORC1.

0.2×10^6^ cells were lysed in SDS Sample buffer, separated on 8% SDS-PAGE gels and transferred to a nitrocellulose membrane. Immunoblots were stained overnight at 4°C with primary anti-ORC1 (1:1000 TBST/5% milk; Abcam, ab85830). Immunoblots were then incubated in secondary goat anti-rabbit (1:1000 TBST/5% milk; Invitrogen, 32460) for 1 hr at room temperature. Immuno-reactive proteins were detected using the Pierce^®^ ECL system (Thermo Scientific #32106).

#### H2A.Z.

Whole-cell lysates from approximately 0.2×10^6^ cells per sample were separated on 13% SDS-PAGE gels and transferred to a nitrocellulose membrane. The membrane was blocked with TBST/5% BSA for 4 hr. Immunoblots were stained overnight at 4°C with the primary anti-H2A.Z (1:2000; Abcam ab4174). The immunoblots were then incubated with secondary goat anti-rabbit (1:4000; Invitrogen 32460) for 1 hr at room temperature with TBST/5% BSA. Immuno-reactive proteins were detected using the Pierce^®^ ECL system (Thermo Scientific #32106).

#### c-MYC.

Cells (0.2×106) were lysed using SDS Sample buffer, and the lysates were then subjected to separation on an 8% SDS-PAGE gel, followed by transfer to a nitrocellulose membrane. The membrane was incubated overnight at 4°C with the primary antibody mAb c-MYC (dilution 1:1000 in TBST/5% milk; Cell Signaling Technology, D84C12). Subsequently, the membrane was incubated with secondary goat anti-rabbit antibodies (dilution 1:1000 in TBST/5% milk; Invitrogen, 32460) for 1 hr at room temperature. Immuno-reactive proteins were visualized using the Pierce^®^ ECL system (Thermo Scientific #32106).

### Electrophoretic gel mobility shift assays (EMSAs)

DNA and RNA oligonucleotides were end-labeled with [γ−^32^P] ATP (Perkin Elmer) and T4 polynucleotide kinase (New England Biolabs). Reactions were incubated at 37 °C for 1 hr and then passed through G-25 spin columns (GE Healthcare) according to the manufacturer’s instructions to remove unincorporated radioactivity. Labeled samples were gel-purified on 10% polyacrylamide gels. Binding reactions were carried out in 10 μl volumes in the following buffer: 5 mM Tris pH 7.4, 5 mM MgCl_2_, 1 mM DTT, 3% v/v glycerol, 100 mM NaCl. 5 nM of full-length purified ORC1 (Cusabio Catalog N. CSB-BP621667HU(A4)) and H2A.Z (Active Motif, Cat. No. 81072) proteins were incubated with 1.1 nM of ^32^P-labeled single-stranded RNAs. All reactions were assembled on ice and then incubated at room temperature for 30 min in Binding Buffer (HEPES-NaOH 25mM, Magnesium Acetate 10mM, Sodium Glutamate 100mM, EDTA 0,1 mM, DTT 1mM, ATP 3mM, RNase 20U, Glycerol 5%) ^27^. Samples were loaded onto 6% native polyacrylamide gels (0.5xTBE) at 4°C and ran for 3 hr at 140 V. All gels were fixed (20% Methanol, 10% Acetic acid), dried and exposed to X-ray film. RNA oligonucleotides are listed in Supplementary Table B.

### Chromatin immunoprecipitation (ChIP) and nascent chromatin immunoprecipitation (nas-ChIP)

ChIP was performed as follows. Cells were crosslinked with 1% formaldehyde for 10 min at room temperature. Pellets of 1×10^6^ cells were used for immunoprecipitation as previously described ^22,24^ and lysed for 10 min on ice and chromatin fragmented using a Branson 250 digital sonicator. Each ChIP was performed with 4 μg of antibody, incubated overnight at 4°C. A 50/50 slurry of protein A and protein G Dynabeads was used to capture enriched chromatin, which was then washed before reverse-crosslinking and proteinase K digestion at 65°C. Immunoprecipitated DNA was analyzed by ChIP-PCR analysis and ChIP-seq. The following antibodies were used for ChIP: H2A.Z (Abcam ab4174, lot GR3176820–1) and IgG (Abcam ab171870). Fold enrichment was calculated using the formula 2^(−ΔΔCt(ChIP/non-immune serum))^.

Primer sets used for ChIP are listed in Supplementary Table A.

### RNA immunoprecipitation (RIP)

The RIP protocol was conducted following the established procedure ^24^ with slight modifications. In summary, 1. 6×10^6^ K562 cells were crosslinked with 1% freshly made formaldehyde solution (50 mM HEPES-KOH; 100 mM NaCl; 1 mM EDTA; 0.5 mM EGTA; 11% formaldehyde) for 10 min at room temperature. 2. Crosslinking was stopped by adding 1/10^th^ volume of 2.66 M Glycine, kept for 5 min at room temperature and 10 min on ice. 3. Cell pellets were washed twice with ice-cold PBS (freshly supplemented with 1 mM PMSF). 4. Cell pellets were resuspended in cell lysis buffer (volume = 4 mL), 1x Buffer (10 mM Tris pH 7.4; 10 mM NaCl; 0.5% NP-40), freshly supplemented with protease inhibitors (protease inhibitor cocktail: Roche Applied Science, Cat. No. 1836153, 1 tablet/375 μL H_2_O; add as x100), 0.1 mM PMSF, and 0.2 mM vanadyl complex (NEB). 5. Cells were incubated at 0°C for 10–15 min and homogenized in a Dounce (10 strokes pestle A and 40 strokes pestle B). 6. Nuclei were recovered by centrifugation at 2,000 rpm for 10 min at 4°C. 7. Nuclei were resuspended in 3 ml of 1x Resuspension Buffer (50 mM HEPES-NaOH, pH 7.4; 10 mM MgCl_2_) supplemented with 0.1 mM PMSF and 0.2 mM vanadyl complex. 8. DNase I treatment (250 U/ml) was performed for 30 min at 37°C, and EDTA (final concentration 20 mM) was added to stop the reaction. 9. Resuspended nuclei were sonicated once for 20 s (1 pulse every 3 s) at 30% amplitude (Branson Digital Sonifer, Danbury, CT).

**Immunoprecipitation** was performed as follows: 1. Before preclearing, the sample was adjusted with 1% Triton X-100; 0.1% sodium deoxycholate; 0.01% SDS; 140 mM NaCl; Protease inhibitors; 0.2 mM vanadyl complex; 0.1 mM PMSF. 2. Preclearing step: ~ 50 μL magnetic beads (Protein A or G Magnetic Beads; #S1425S or #S1430S NEB) were added to the sample, and incubation was carried out for 1 hr on a rocking platform at 4°C. 3. Beads were removed in the magnetic field. 4. The sample was then divided into aliquots according to different conditions and antibodies of interest: (1) H2A.Z (Abcam, ab4174), ORC1 (Abcam, ab85830), preimmune serum: IgG (ab171870); (v) no antibody, no serum (input). 5. 5 μg antibody or preimmune serum was added to the respective aliquot and incubation performed on a rocking platform overnight at 4°C. Input was stored at −20 °C after the addition of SDS to 2% final concentration. Day II. 6. 200 μL of Protein A coated super-paramagnetic beads (enough to bind 8 μg IgG) were added to the samples and incubated on a rocking platform for 1 h at 4°C. 7. Six washes of beads in the magnetic field were performed with immunoprecipitation buffer (150 mM NaCl; 10 mM Tris-HCl, pH 7.4; 1 mM EDTA; 1 mM EGTA pH 8.0; 1% Triton X-100; 0.5% NP-40 freshly supplemented with 0.2 mM vanadyl complex and 0.2 mM PMSF) in a magnetic field. 8. Proteinase K treatment to release DNA/RNA into solution and to reverse the crosslinking was performed in 200 μL of: 100 mM Tris-HCl, pH 7.4; 0.5% SDS for the immunoprecipitated samples and in parallel for the input using 500 μg/mL of Proteinase K at 56°C overnight. 9. Day III. Beads were removed in the magnetic field. 10. Immunoprecipitated RNAs were further purified by extraction with Phenol (pH 4.3) after addition of NaCl (0.2 M final concentration). 11. Ethanol precipitation (in the presence of glycogen); 3 hr at −20°C. 12. The pellet was dissolved in 180 μL H_2_O, heated at 72 °C for 2 min, and immediately chilled on ice. 13. Samples were treated with DNase I (20 units) in the presence of RNase inhibitor at 300 U/ml in x1 buffer # 2 (NEB) at 37°C for 30 min. 14. Phenol (pH 4.3) extraction and EtOH precipitation were repeated. 15. The RNA pellet was dissolved in 50 μL H_2_O and used in qRT-PCR (Primer sets used for RIP-PCR are listed in Supplementary Table A)

### Down-regulation of *c-MYC ANCOR*

#### dCAS9 -mediated stable and inducible downregulation.

K562 and HEK 293 cell lines were maintained in appropriate culture media supplemented with 10% fetal bovine serum (FBS) and 1% penicillin-streptomycin before lentiviral transduction. The guide RNA (gRNA) targeting the *c-MYC ANCOR* in a region located 1.4kb upstream of the *c-MYC* origin was designed using the online tool provided by the Broad Institute (https://portals.broadinstitute.org/gpp/public/analysis-tools/sgrna-design), and an initial guide screening was conducted. To implement CRISPR interference (CRISPRi), the lentiviral vector pdCAS9-humanized (code 44246) with an engineered mCherry fluorescent signal was acquired from Addgene and transduced into K562 cells, following the protocol outlined in ^31^. Four lentiviral vectors (Cellecta-pRSGT16-sgRNA containing guide RNAs and no-targeting control guide (gRNA-1, gRNA-2, gRNA-3, ntRNA)) were purchased from Collecta, packaged into second-generation lentiviral particles, and transduced into K562 cells stably expressing dCas9 using Lipofectamine 3000 transfection reagent (Thermo Fisher Scientific) according to the manufacturer’s instructions. In the Tet-inducible system, all guide RNAs were cloned under the control of a Tet-inducible promoter. In parallel, a non-targeting RNA control was also transfected. To induce the expression of gRNAs, cells were treated with a single addition of 5 μM doxycycline. For assessing guide RNA-2 expression in cells, custom primers and TaqMan probe were designed.

The lists of the guides and the primers are provided below:

**Table:** 

**Guide RNA sequences**	
scramble	GCACTCACATCGCTACATCAGGG
gRNA1	CTGGATGTCAACGAGGGCGGGGG
gRNA2	ACTTTCGCAAACCTGAACGCGGG
gRNA3	CAGGCCTTTGCCGCAAACGCGGG

**Table:** 

**Primer set sequences**	
Forward Primer_gRNA2	5’-CGACTTTCGCAAACCTGAAC-3’
Hybridization probe_gRNA2	5’-TGCTGGAAACAGCATAGCAA-3’
Reverse Primer_gRNA2	5’-ACTCGGTGCCACTTTTTCAA-3’
Forward Primer_NTgRNA	5’-GCAGTCGTTCGGTTGATATG-3’

#### ASO/FANA-ASO antisense oligonucleotide-mediated downregulation.

ASOs targeting c-MYC *ANCOR* and no-targeting scrambled control were purchased from FANA AUM biotech (proprietary sequence). They were added to cell cultures at 10–20 μM concentration. Downregulation was monitored, and cell samples were collected at 48, 72, and 96 hr after ASO treatment for subsequent qPCR analysis.

### Upregulation of *c-MYC ANCOR*

#### dCAS9_VP64 -mediated upregulation.

A lentiviral vector (pLenti-U6-sgRNA-PGK-Neo) encoding the designed gRNA was synthesized by Applied Biological Materials Inc. pMD2.G, pCMV delta R8.2, and the lentiviral vectors were transfected into 10 million 293T cells using TransIT-LT1 reagent (Mirus). Supernatants were harvested at 48 and 72 hr after transfection. Virus was concentrated 100 times by Lenti-X Concentrator (Takara) after filtering through a 0.45 μm syringe filter and resuspended in DMEM and stored at −80°C. dCas9-VP64 (Addgene, plasmid #61425) lentiviral transduction was performed in the presence of hexadimethrine bromide (final concentration 8 μg/ml) in HEK 293 cells. Blasticidin (10 μg/ml) was added to the cultures 2 days after infection. Stably expressing dCas9-VP64 cells were subsequently transduced with *c-MYC ANCOR* gRNA2 or scrambled control. G418 (500 μg/ml) was added to the cultures 2 days after infection. Resistant clones were selected and screened to assess *DNA* replication and *c-MYC ANCOR* levels.

The gRNA sequences are shown below:

**Table:** 

***c-MYC* locus**	
scramble	5'- GCACTCACATCGCTACATCAGGG -3'
guideRNA1	5'- CACCGCCCCCTACTCTAGCACTGAA -3'
guideRNA2	5'- CACCGGTGGCCCGAGCTGCATCTCG -3'
guideRNA3	5'- CACCGACAGCCCCTAATGACTCTCC -3'

### DNA fiber analysis

K562 cells were cultured and maintained in appropriate growth media until reaching a cell number of 15 million. Synchronization in the S phase of the cell cycle was achieved using the Double Thymidine block method, following established procedures ^36^. Additionally, DRB treatments were employed to induce an early S-phase block in transcription, as described in the literature ^24^. The DNA fiber assay was performed as previously described ^48^. Briefly, cells were sequentially pulse labeled with 25 μM CldU followed by 250 μM IdU for 30 min at 37°C. Untreated cells were used as controls. Cells were washed with ice-cold PBS, trypsinized, and resuspended at 2.5×10^6^ cells/mL concentration in cold PBS. 2 μl of cell suspension was gently placed on a glass slide, and after 2–3 min of incubation, lysis buffer (200 mM Tris-HCl; pH 7.5, 50 mM EDTA and 0.5% SDS) was added, followed by 2 min of incubation at room temperature. The slides were tilted at an angle (15–45°) to allow the fibers to spread along the slide. After drying, the slides were fixed in methanol-acetic acid (3:1) for 15 min at −20°C, denatured in 2.5 M HCl for 30 min, and blocked in 5% BSA in 1X PBS for 20 min (blocking buffer). Primary anti-BrdU antibodies specific for CldU (1:15) and IdU (1:15) were applied for 1 hr at room temperature followed by PBS wash. Slides were then stained with secondary antibodies, anti–rat Alexa Fluor 488 (1:15) and anti–mouse Alexa Fluor 568 (1:15), for 1 hr at room temperature. Slides were mounted with ProLong Antifade Mountant (Thermo Fisher Scientific #P10144) and imaged on a Carl Zeiss Axio Imager M2 microscope. Labeled replication structures were identified, and the track length was measured using ImageJ. The replication event initiation and termination were also recorded and quantified. We used Student’s t-test with a two-tailed distribution for p-value calculation.

### Single molecule analysis of replicated DNA (SMARD) analysis

When the cells reached 70–80% confluence, they were synchronized in S phase using the Double Thymidine block method ^36^, and DRB treatments were employed to induce block of early S-phase transcriptional activities as described in the literature ^24^. The cells were then incubated at 37°C for 4 hr in the presence of 25 μM IdU (Sigma-Aldrich, St. Louis, MO). After washing cells with PBS, hESC medium with 25 μM CldU (Sigma-Aldrich, St. Louis, MO) was added to the cultures, and the cells were incubated for an additional 4 hr. The cells were harvested with Accutase for hESC or Trypsin for mammalian epithelial cells. Following centrifugation, the cells were resuspended at 3 × 10^7^ cells per ml in PBS. 1% InCert agarose (Lonza Rockland, Inc, FMC, lot 221291) in PBS was added to an equal volume of cells at 42°C. The cell suspension was pipetted into a chilled plastic mold with 0.5- by 0.2-cm wells with a depth of 0.9 cm for preparing DNA gel plugs. The gel plugs were allowed to solidify on ice for 30 min. Cells were lysed in buffer containing 1% *n*-laroylsarcosine (Sigma Aldrich), 0.5 M EDTA, and 20 mg/ml proteinase K. The gel plugs remained at 50°C for 64 hr and were treated with 20 mg/ml proteinase K every 24 hr. Gel plugs were then rinsed several times with Tris-EDTA (TE) and once with phenylmethanesulfonyl fluoride. The plugs were washed with 10 mM MgCl_2_ and 10 mM Tris-HCl (pH 8.0). The genomic DNA in the gel plugs was digested with 40 units of *PmeI* (at 37°C overnight. The digested gel plugs were rinsed with TE and cast into a 0.7% SeaPlaque GTG agarose gel (Lonza Rockland, Inc.). A gel lambda ladder PFG marker and yeast chromosome PFG marker (both from New England BioLabs, Inc.) were cast next to the gel plugs. PCR was performed to determine the appropriate positions of DNA on the pulsed-field electrophoresis gel, and the gel piece was cut and melted at 72°C for 20 min. β-Agarase enzyme (1 unit per 50 μl of agarose suspension) was carefully added to digest the agarose and incubated at 45°C for a minimum of 2 hr. The resulting DNA solutions were stretched on 3-aminopropyltriethoxysilane-coated glass slides. The DNA was pipetted along one side of a coverslip that had been placed on top of a silane-treated glass slide and allowed to enter by capillary action. The DNA was denatured with sodium hydroxide in ethanol and then fixed with glutaraldehyde. The slides were hybridized overnight with a biotinylated probe (the blue bars diagrammed on the maps indicate the positions of the probes used). The following day, the slides were rinsed in 2× SSC buffer with 1% SDS and washed in 40% formamide solution containing 2 × SSC at 45°C for 5 min and rinsed in 2 × SSC-0.1% IGEPAL CA-630. Following several detergent rinses (4 times in 4× SSC-0.1% IGEPAL CA-630), the slides were blocked with 1% BSA for at least 20 min and treated with Avidin Alexa Fluor 350 (Invitrogen Molecular Probes) for 20 min. The slides were rinsed with PBS containing 0.03% IGEPAL CA-630, treated with biotinylated anti-avidin D (Vector Laboratories) for 20 min, and rinsed again. The slides were then treated with Avidin Alexa Fluor 350 for 20 min and rinsed again, as in the previous step. The slides were incubated with the IdU antibody, mouse anti-bromodeoxyuridine (Becton Dickinson Immunocytometry Systems), the CldU antibody, monoclonal rat anti-bromodeoxyuridine (anti-BrdU) (Accurate Chemical and Scientific Corporation), and biotinylated anti-avidin D for 1 hr. This was followed by incubation with Avidin Alexa Fluor 350 and secondary antibodies, Alexa Fluor 568 goat anti-mouse IgG (H+L) and Alexa Fluor 488 goat anti-rat IgG (H+L) (both from Invitrogen Molecular Probes), for 1 hr. After a final PBS/ IGEPAL CA-630 rinse, the coverslips were mounted with ProLong Gold Antifade Mountant. A fluorescent microscope (Axioscop 2 M2 with Plan Apochromat 63×/1.4 NA oil differential interference contrast objective; Carl Zeiss) with a camera (CoolSNAP HQ; Photometrics) was used to detect the nucleoside incorporation into the DNA molecules. Images were processed with Photoshop CS5 software. Image processing involves alignment of the DNA molecules according to the FISH probes, adjusting of the contrast, and removing unspecific background signal. The molecules presented are the complete dataset.

For the *FXN* gene locus, biotinylated probes were prepared from Fosmids G248P83100G6 and G248P85473G3 that were selected using the public gene database (http://genome.ucsc.edu/). To analyze the replication fork progression, the % molecules with IdU incorporation moving across the *FXN* locus were quantified. The segment was divided into 5kb fragments, and replication fork per segment was counted. The percentage of molecules with IdU incorporation was calculated for the 5kb interval.

### Modeling and molecular dynamics simulations

The RM9 and dRM9 models were built by using the MC-sym/MC-fold and the 3dnus procedures ^49^. The coordinates of the Orc1 protein as deposited in the alphaFold protein structure database were downloaded and used as a starting model of the entire protein because none of the experimental solved structures available in the pdb data bank contain the full-length protein coordinates. The created theoretical models of the interacting systems were individually subjected to molecular dynamic simulations to refine the structures and to explore the conformational dynamics, following validated protocols. In detail, 100 ns of simulation time was used to minimize RM9 and dRM9 systems, assessing the validity of their initial conformations. For the Orc1 alphaFold predicted structure, we applied three diverse velocity simulations to refine the model and better explore the Orc1 conformational ensembles, which contain a high level of flexibility. Indeed, the high prevalence of disordered regions, particularly between the N- (residues 1–479) and the C- (residues 480–861) segments suggest that the protein can explore various equally probable states. No experimental data were available to define the relative orientations between these domains. We thus extracted from each of the three trajectories a representative ORC1 conformation (namely orc-1a, orc-1b and orc-1c) and used each as a target to dock the RM9 and dRM9 sequences. The pdb 7JPS (PubMed ID: 32808929) structures were used as a template to accommodate the RNA sequence. In detail, orc1a orc1b and orc1c states were structurally overlapped to the Orc1 subunit of 7jJPS (chain A) pdb, and the RNA sequence models were positioned to replace the region occupied by the DNA molecules of the same pdb that was used as a template for the docking.

### Bioinformatics

#### RNA sequencing (RNA-Seq).

Strand-specific transcriptome libraries were constructed on BGI Genomics platform (DNBSEQ). Pipeline of the experiment: 1) mRNA and non-coding RNAs were enriched by removing rRNA; 2) by using the fragmentation buffer, the mRNA and non-coding RNAs were fragmented into short fragments (about 200~700 bp); 3) the first-strand cDNA was synthesized by random hexamer-primer using the fragments as templates; 4) buffer, dNTPs, RNase H and DNA polymerase I were added to synthesize the second strand cDNA; 5) the double-stranded cDNA was purified with a QiaQuick PCR extraction kit and then used for end-polishing; 6) sequencing adapters were ligated to the fragments, and then the second strand was degraded using Uracil-N-Glyosylase); and 7) the fragments were purified by Agarose gel electrophoresis and enriched by PCR amplification. Libraries were paired-end sequenced on Hi-Seq-4000 Illumina. Raw reads were analyzed using a pipeline for RNA-seq analysis - Visualization Pipeline for RNA-seq analysis (VIPER) based on workflow management system Snakemake (https://github.com/hanfeisun/viper-rnaseq). The read alignment to hg19 reference genome was performed using STAR aligner (2.7.0f) with default parameters. Gene expression (FPKM values) was quantitated with Cufflinks (v2.2.1). Bedtools (v2.27.1) genomecov and bedGraphToBigWig v 4 were used to generate bigwig files.

#### RIP-sequencing.

RNAs were processed for sequencing as described by Di Ruscio *et al*. with minor adaptations^24^. RNA samples were depleted of ribosomal RNA with Ribo-ZeroTM Magnetic Gold Kit (cat. # MRZG126 Epicentre). Libraries were constructed on BGI Genomics platform (DNBseq, RIPseq). Pipeline of the experiment: 1) RNA was enriched; 2) the first-strand cDNA was synthesized by random hexamer-primer using the fragments as templates; 3) second-strand synthesis and ER/A-tailing were performed; 4) ligation was accomplished with an overhanging T at the 3’ end of the bubble adapter; 5) the ligated products were amplified by PCR with specific primers; 6) single-strand separation was achieved by thermal denaturation and a bridge primer was used for circularization of the single-stranded DNA to obtain a single-stranded circular DNA library; 7) for sequencing of amplified products, DNA Nanoballs (DNBs) were used for paired-end sequencing on Hi-Seq-4000 Illumina. Raw data with adapter sequences or low-quality sequences were filtered. This step was completed by SOAPnuke software developed by BGI. SOAPnuke software filter parameters: -n 0.001 -l 20 -q 0.4 -A 0.25 --cutAdaptor -Q 2 -G --minLen 100. Raw reads were analyzed using the pipeline, CHromatin enrIchment ProcesSor Pipeline (CHIPS), based on the workflow management system Snakemake (https://github.com/liulab-dfci/CHIPS) where the read alignment to the hg19 reference genome was performed using bwa mem aligner (0.7.15-r1140) with default parameters. The MACS2 algorithm (2.2.7.1) was used to call peaks, and both MACS2 (2.2.7.1) and bedGraphToBigWig (v 4) were used to generate bigwig files. Read counts for downstream analyses were quantified using the bioliquidator/bamliquidator tool from Docker image (https://hub.docker.com/r/bioliquidator/bamliquidator). Genomic regions were annotated using Hypergeometric Optimization of Motif EnRichment tool (HOMER v3.12).

#### ChIP Sequencing.

ChIP-libraries were constructed on the BGI Genomics platform (DNBseq, CHIPseq). ChIP library construction pipeline: 1) end repair and adapter ligation (filling the ends of IP/Input DNA followed by 5’ phosphorylation. A 3’ sticky-end with an overhanging A was formed, which could ligate with an overhanging T at the 3’ end of the bubble adapter); 2) PCR amplification (PCR amplification of the ligated products with specific primers); 3) single-strand separation and circularization (separation of PCR products into single-strands by thermal denaturation. Then circularization of the single-stranded DNA through a bridge primer to obtain single-stranded circular DNA library); 4) for sequencing of amplified products, DNA Nanoballs (DNBs) were used for paired-end sequencing on Hi-Seq-4000 Illumina. Raw data with adapter sequences or low-quality sequences were filtered. We first went through a series of data processing to remove contamination and obtain valid data. This step was completed by SOAPnuke software developed by BGI. SOAPnuke software filter parameters: -n 0.01 -l 20 -q 0.4 --cutAdaptor -Q 2 -G --polyX 50 --minLen 100. Raw reads were analyzed using a pipeline CHromatin enrIchment ProcesSor Pipeline (CHIPS) based on the workflow management system Snakemake (https://github.com/liulab-dfci/CHIPS) where the read alignment to the hg19 reference genome was performed using bwa mem aligner (0.7.15-r1140) with default parameters. MACS2 algorithm (2.2.7.1) was used to call peaks, and both MACS2 (2.2.7.1) and bedGraphToBigWig (v 4) were used to generate bigwig files. Read counts for downstream analyses were quantified using the bioliquidator/bamliquidator tool from Docker image (https://hub.docker.com/r/bioliquidator/bamliquidator). Genomic regions were annotated using Hypergeometric Optimization of Motif EnRichment tool (HOMER v3.12.

#### Graphics.

Genomic heatmaps were generated using computeMatrix and plotHeatmap scripts from deeptools (v3.5.1). All Boxplots were generated using the “ggplot2” R package (https://ggplot2.tidyverse.org). ScatterDensity plots were generated using the smoothScatter R function (https://www.rdocumentation.org/packages/graphics/versions/3.6.2). Genome browser pictures were generated using the “WashU Epigenome Browser” (https://github.com/lidaof/eg-react/).

### Statistical analysis

All the statistical analyses were performed using the R suite (https://www.r-project.org/). Comparisons of the distributions corresponding to ChIP-Seq, RIP-Seq and RNA-Seq cumulative read count signals were performed using the Mann Whitney U test or Wilcoxon signed rank test, with a confidence interval of (℘ < 0.05). For the statistical evaluation of the HAT assay, we applied two-way ANOVA with multiple comparisons. GraphPad Prism Software was used. Values of p≤ 0.05 were considered statistically significant.

## Supplementary Material

1

## Data Availability

Sequencing data are available on the gene omnibus database under the accession ID number **GSE241684**, using the secure token **qtelsuwwfnqfhef**. Computational simulations, including predictions of molecular structures, have been deposited in ModelArchive under the following projects: Project: **orc1a-dRM9 | ma-58rip** https://www.modelarchive.org/doi/10.5452/ma-58rip Pass: **PYpBM1K8GF** Project: **orc1a-RM9 | ma-mrb6z** https://www.modelarchive.org/doi/10.5452/ma-mrb6z Pass: **e3NzM2ZgtB** Project: **orc1b-dRM9 | ma-33nek** https://www.modelarchive.org/doi/10.5452/ma-33nek Pass: **1Sf0pa962a** Project: **orc1b-RM9 | ma-pggyf** https://www.modelarchive.org/doi/10.5452/ma-pggyf Pass: **MKMRW9RcjN** Project: **orc1c-dRM9 | ma-drb5v** https://www.modelarchive.org/doi/10.5452/ma-drb5v Pass: **fPL9qyuzzU** Project: **orc1c-RM9 | ma-59js0** https://www.modelarchive.org/doi/10.5452/ma-59js0 Pass: **esx9E1IiOu**

## Highlights

- *ANCORs* (RN***A***s a***N*C**horing ***OR***C1) are *initiators* of DNA replication origins in human cells
- *ANCORs* control the engagement of ORC1 and H2A.Z, allowing the formation of an active origin
- *ANCORs* lead to the generation of active replication origins
