# Supplementary material for: RNAs anchoring replication complex control initiation and firing of DNA replication
Source: Res Sq. 2025 Jan 28:rs.3.rs-5723221. Preprint. [Version 1] doi: 10.21203/rs.3.rs-5723221/v1 (PMC11838740; doi:10.21203/rs.3.rs-5723221/v1)
Supplement: 1 [file NIHPPrs5723221v1-supplement-1.pdf]

SUPPLEMENTAL INFORMATION

Supplementary Table A. Primer sequences used for chromatin and RNA immunoprecipitation qPCR (*c-MYC* locus)

Forward mychip1	5'-GGCTAATCCTCTATGGGAGTCTGT C-3'
Reverse mychip2	5'- TTTCTGAATACTAGTGAAAGTGCA-3'
Forward mychip3	5'- TCAGAAAAAATTGTGAGTCAGTGA -3'
Reverse mychip4	5'- TTGTGGACCGAGCCGGGGGAGTCA -3'
Forward mychip7	5'- ACAGGCAGACACATCTCAGGGCTA -3'
Reverse mychip8	5'- ATAGGGAGGAATGATAGAGGCATA -3'
Forward mychip9	5'- CTACACTAACATCCCACGCTCTGA -3'

Reverse mychip10	5'- AACCGCATCCTTGTCCTGTGAGTA -3'
Reverse mychip18	5'- CCCCAGTTACCATAACTACTCTGA -3'
Forward mychip19	5'- GGATCGGGGTAAAGTGACTTGTCA -3'
Reverse mychip 20	5'- GCGGCTGCGGAGCGATCTGGCTCA -3'
MYC11-F	5′-TATCTACACTAACATCCCACGCTCTG-3′
MYC11-R	5′-CATCCTTGTCCTGTGAGTATAAATCATCG-3
Alu primer set (Alu115); Forward	5’-CCTGAGGTCAGGAGTTCGAG-3’
Alu primer set (Alu115); Reverse	5’-CCCGAGTAGCTGGGATTACA-3’

Supplementary Table B. RNA/DNA oligonucleotides used in EMSA assay:

RM9	5’-GAGAACGCACUGCGCGCCCACCGCCACGCCACGCGCGUAC-3’
MYC1	5’-CAGCCGCCCACUUUUGACAGGCCUGGGCGGGCUUCGCUUA-3’
dRM9	5’-GTACGCGCGTGGCGTGGCGGTGGGCGCGCAGTGCGTTCTC-3’

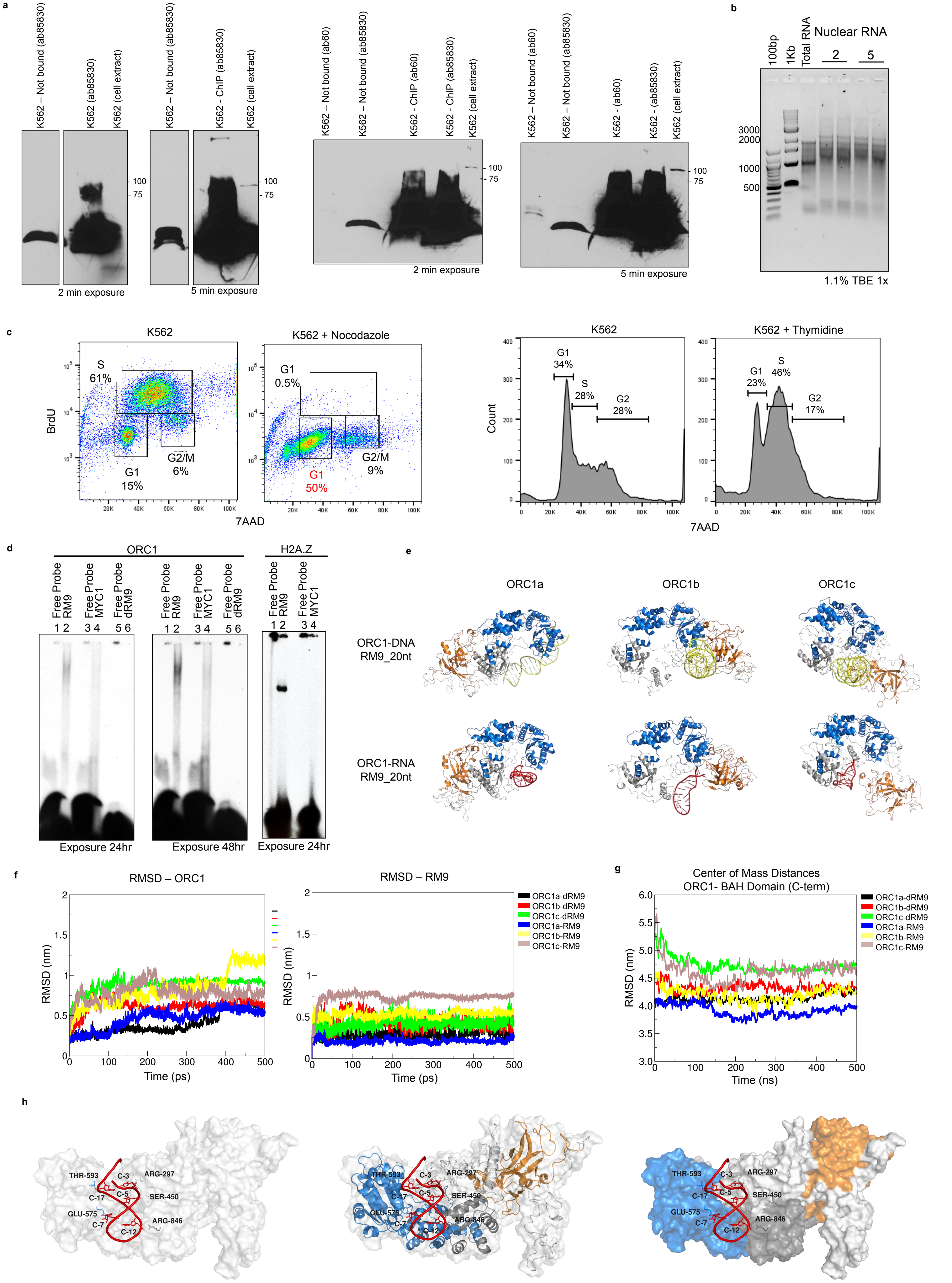

Ummarino *et al.* Supplementary Figure 1

Supplementary Figure 1| Characterization of ORC1-G1/S phase RNA interaction.

a. Validation by western blot analyses of two independent ORC1 antibodies using either K562 whole cell extract or ORC1 immunoprecipitates; **b** Electrophoresis analysis shows the integrity of total RNA and nuclear RNA fractions run on 1.1% Agarose gel in 1x Tris-Borate-EDTA (TBE) buffer; **c** Assessment of the cell cycle synchrony by FACS analysis of K562 synchronized in phase G1 (using Nocodazole 15 mM, 24 hr) and phase S (using double Thymidine block (2.5 mM, 18 hr) **d**. RNA and DNA Electrophoretic Mobility Shift Assays (EMSA) show binding of ORC1 to RM9 (RNA), but not MYC1 or the homologous DNA sequence dRM9 (left and middle panels indicate different exposure times). RM9 interacts with ORC1 unlike MYC1 (right panel); **e**. Frontal view of three apo ORC1 states (ORC1a, ORC1b, and ORC1c) showing distinct conformations, mainly differing in the relative orientation between the N- and C- ORC1 regions and the BAH domain. Orange: ORC1-BAH domain (residues 1-200); marine: ORC1 disordered region and AAA+ domain (residue 201-782); gray: orc1-WHD domain (residues 783-861); red and yellow: RM9 and dRM9, respectively; **f**. Root Mean Squared Deviation (RMSD) profiles of the ORC1-RM9 complexes during extended molecular dynamics (MD) simulations. The ORC1a-RM9 complex exhibits the smallest changes from the initial state. (**Left panel**) The RSMD profiles computed using only the C-alpha (C-a) atoms of each ORC1 state residue along 500 ns of six complex MD simulations with the respect to the starting run state. Orc1a-dRM9, black (mean: 0.6 nm, SD: 0.15); orc1b-dRM9, red (mean: 0.6 nm, SD 0.09); orc1c-dRM9, green (mean: 0.9 nm, SD: 0.09); orc1a-RM9, blue (mean: 0.5 nm, SD: 0.13); orc1b-RM9, yellow (mean: 0.8 nm, SD: 0.21); and orc1c-RM9, brown (mean 0.9 nm, SD 0.11). (**Right panel**) The RSMD profiles computed using only the C1' atom of either RNA or DNA sequence along 500 ns of six complex MD simulations with the respect to the starting state. Orc1a-dRM9, black (mean: 0.3 nm, SD: 0.05); orc1b-dRM9, red (mean: 0.4 nm, SD 0.09); orc1c-dRM9, green (mean: 0.4 nm, SD: 0.07); orc1a-RM9, blue (mean: 0.2 nm, SD: 0.03); orc1b-RM9, yellow (mean: 0.5 nm, SD: 0.04); and orc1c-RM9, brown (mean 0.7 nm, SD 0.07); **g**. Distances between the center of mass of the ORC1-BAH domain (residues 1-200) and the C-terminal region (200-861) during the entire simulation. Orc1a-dRM9: black; orc1b-dRM9: red; orc1c-dRM9, green; orc1a-RM9: blue; orc1b-RM9: yellow; and orc1c-RM9: brown; **h**. Hydrogen bond interactions between RM9 and ORC1a residues, indicating the key residues involved in the interaction. ARG297, SER450, GLU575, THR593, and ARG846 predominantly mediate the connection between ORC1a and RM9 during the simulation.

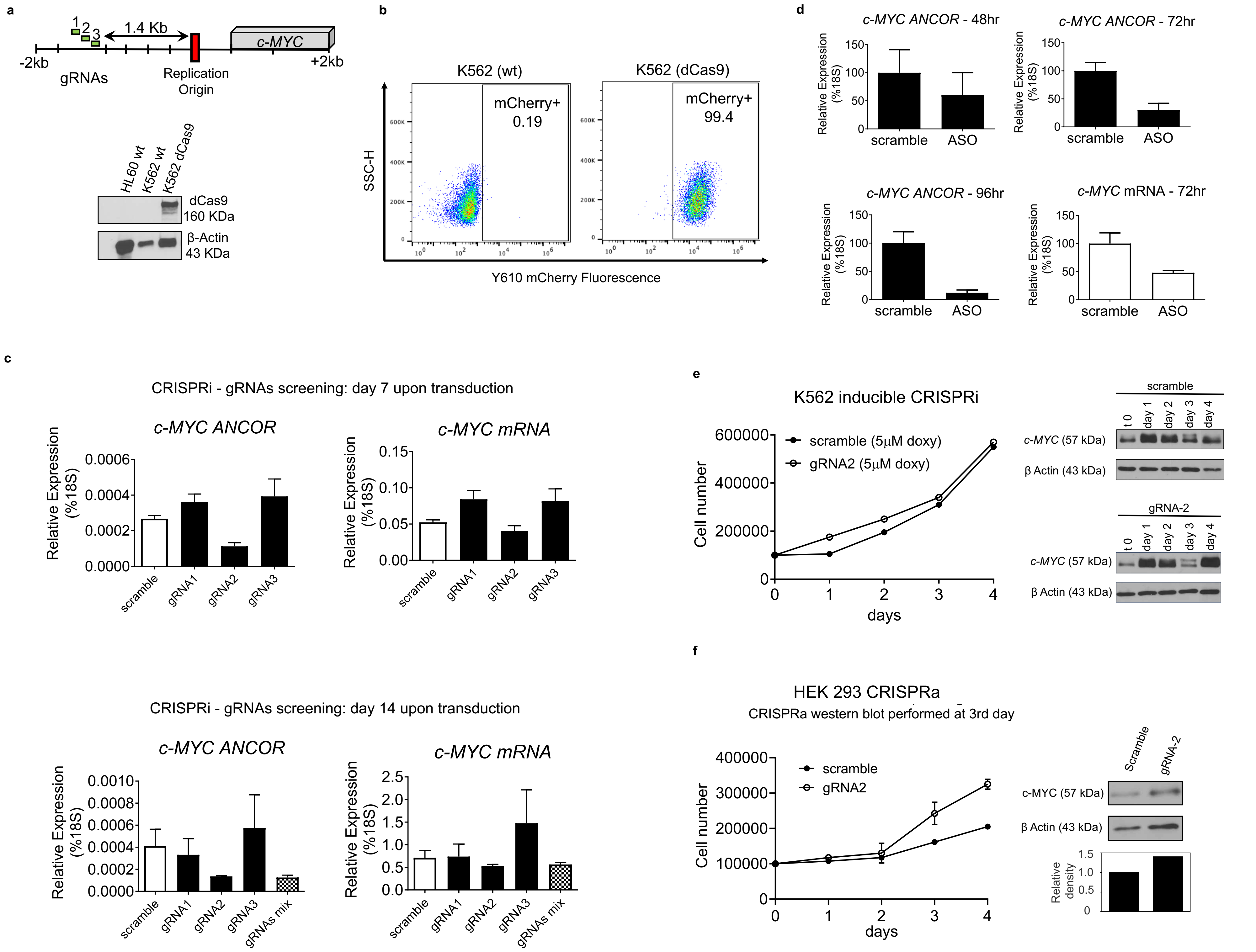

Ummarino *et al.* Supplementary Figure 2

Supplementary Figure 2| *c-MYC ANCOR* loss and gain of function by CRISPRi and CRISPRa.

a. Schematic of *c-MYC* locus showing gRNA targets and expression of dCas9 protein levels in K562 cells assessed by western blot analysis; **b**. Flow cytometry analysis showing percentage of K562 cells expressing dCa9-mCherry (99.4%); **c**. qRT-PCR screening of the effect of non-inducible gRNAs on *c-MYC ANCOR* and *c-MYC* mRNA levels, 7 and 14 days upon viral transduction in K562-dCa9-mCherry.; **d**. ASOs targeting *c-MYC ANCOR* and a non-targeting control were delivered into K562 at a concentration between 15 µM. Samples collected at 48, 72 and 96 hr were used for qRT-PCR. Downregulation of *c-MYC ANCOR* was observed starting from 48 hr, lasting until 96 hr, and was associated with a significant reduction in *c-MYC mRNA* levels **e**. Growth curve of K562 dCas9-mCherry, expressing the inducible gRNA-2 or the scramble control sequence. No changes in cell number were observed upon the induction of gRNAs by doxycycline in the first 4 days (left panel). Immunoblot analyses of c-MYC levels across a 4-day time course were comparable between the gRNA-2 and scramble control transduced cells (right panel); **f**. Growth curve of HEK 293 cells stably expressing dCas9-VP64 with gRNA-2 or the scramble control over a 4-day time course. Higher cell count was measured at day 3 in the gRNA-2-transduced cells as compared to the control (left panel) and a parallel increase in c-MYC protein was detected by western blot at day 3.

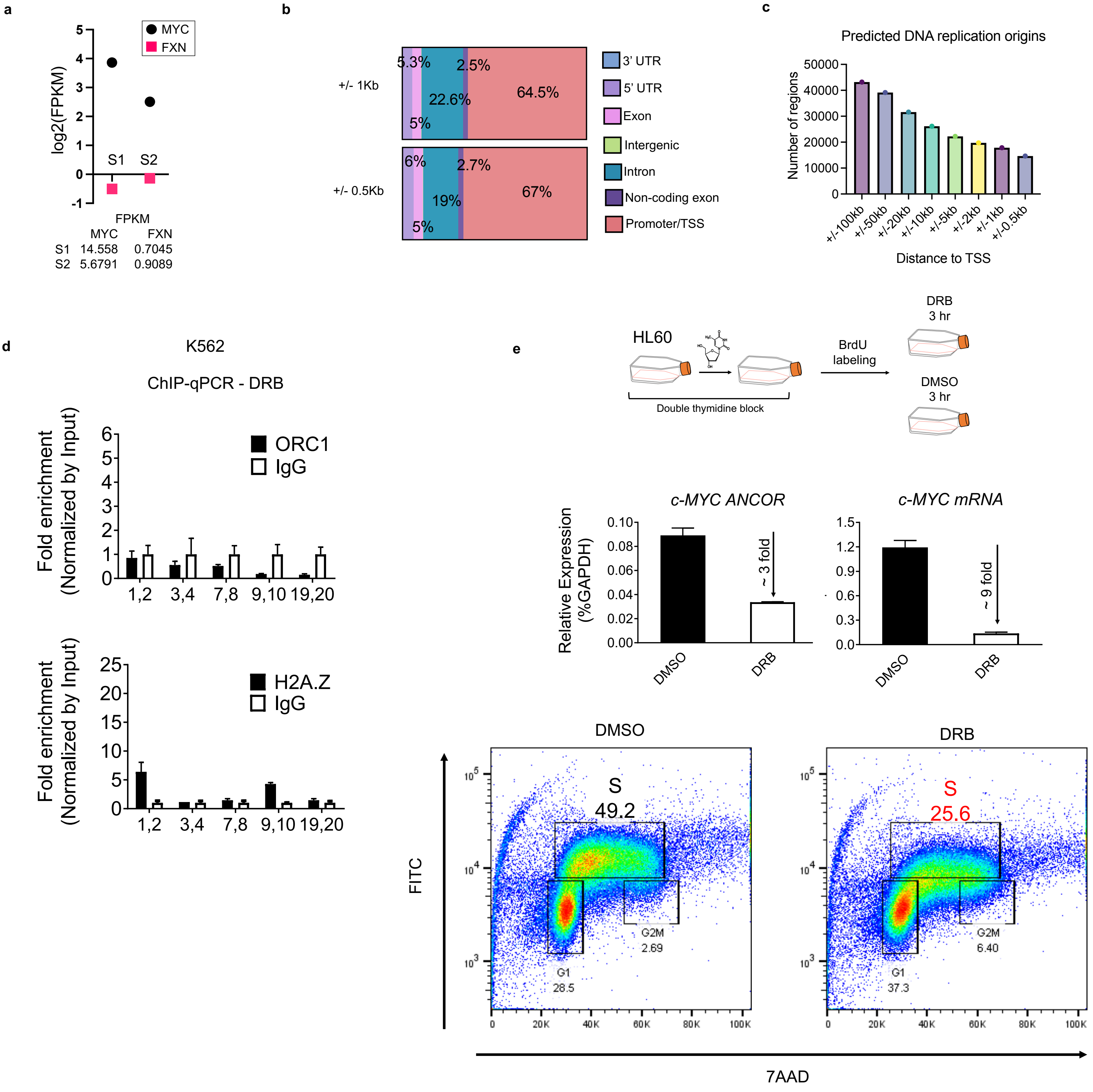

Ummarino *et al.* Supplementary Figure 3

Supplementary Figure 3| G1/S phase RNAs control DNA replication through ORC1 and H2A.Z. **a**. RNA-seq analysis depicting the gene expression levels (FPKM) of *c-MYC* and *FXN* at earlier (S1) and later (S2) stages of S phase; **b**. Annotation of predicted early replication origins across the genome demonstrates that a substantial number are close to the promoters and/or transcription start sites (TSS) of protein-coding genes. The annotation highlights the predicted DNA replication origins positioned within +/-1kb and +/-0.5kb of the TSS; **c**. Histogram graph illustrating the distribution of predicted early DNA replication origins, represented by the number of examined regions plotted against the distance from TSS; **d**. Chromatin immunoprecipitation and qPCR analyses (ChIP-qPCR) following DRB (100 µM) treatment show decreased enrichment of ORC1 and H2A.Z at the *c-MYC* origin (region 9-10). The qPCR analysis was performed with different primer sets, as indicated on the x-axis; **e**. (Upper panel) Schematic representation of HL60 cell synchronization by double thymidine block into G1/S phase, followed by addition of BrdU (3.5 µm) to the growth medium to label nasDNA and either pharmacological inhibition of transcription by DRB (100 µM) or mock treatment with DMSO. Samples were collected 3 hours after DRB and control treatment. (Middle panel) qRT-PCR analysis showing downregulation of *c-MYC ANCOR* and *c-MYC* mRNA expression upon DRB treatment. (Bottom panel) Flow cytometry analysis depicting inhibition of nasDNA synthesis with a drastic reduction in the S phase cell population to nearly 30% in DRB-treated cells as compared to 50% in DMSO-treated cells.

Table S1.

Table 1. Percentage of occurrence of ligand-protein residue connections along the trajectory frames. For each pair, the sum of the hydrogen bonds with occurrence higher than 10% among the trajectory frames between any residue atoms is considered.

dRM9						RM9				
					**orc1a**					
**ARG**	**19**	**DC**	**13**	3'	67	**ARG**	**297**	**C**	**3**	100
**ARG**	**191**	**DG**	**23**	5'	100	**ARG**	**437**	**C**	**19**	61
**SER**	**199**	**DC**	**24**	5'	51	**SER**	**450**	**C**	**5**	100
**SER**	**201**	**DC**	**24**	5'	72	**HIS**	**452**	**G**	**13**	59
**SER**	**201**	**DC**	**25**	5'	47	**THR**	**453**	**G**	**13**	59
**TYR**	**634**	**DC**	**17**	5'	59	**GLU**	**575**	**C**	**7**	75
**LYS**	**643**	**DC**	**19**	5'	75	**HIS**	**577**	**C**	**15**	64
**ARG**	**666**	**DC**	**17**	5'	100	**GLN**	**578**	**C**	**15**	55
**SER**	**668**	**DG**	**28**	3'	67	**LYS**	**591**	**G**	**2**	57
**ARG**	**670**	**DC**	**18**	5'	100	**THR**	**593**	**C**	**17**	78
**LEU**	**673**	**DC**	**27**	3'	44	**ARG**	**846**	**C**	**12**	100
**ARG**	**675**	**DG**	**28**	3'	100					
					**orc1b**					
**ARG**	**24**	**DG**	**23**	5'	66	**LYS**	**25**	G	18	59
**ARG**	**24**	**DG**	**23**	5'	47	**ARG**	**378**	C	11	95
**LYS**	**25**	**DG**	**17**	3'	62	**ARG**	**378**	A	10	65
**TYR**	**28**	**DG**	**16**	3'	45	**ILE**	**379**	C	11	100
**ARG**	**166**	**DG**	**28**	3'	43	**ARG**	**381**	C	9	70
**SER**	**169**	**DC**	**18**	5'	44	**LYS**	**382**	C	7	100
**SER**	**170**	**DC**	**27**	3'	56	**SER**	**383**	C	8	100
**LYS**	**276**	**DC**	**29**	5'	72	**ARG**	**433**	C	5	94
**ARG**	**277**	**DT**	**15**	3'	66	**ARG**	**433**	G	6	83
**ARG**	**277**	**DG**	**16**	3'	43	**ARG**	**444**	C	5	100

**LEU**	**301**	**DG**	**28**	5'	100	**ARG**	**444**	G	4	71
**SER**	**302**	**DG**	**28**	5'	99	**LYS**	**448**	G	2	45
**TYR**	**303**	**DG**	**28**	5'	95	**SER**	**450**	C	3	60
**ARG**	**433**	**DC**	**25**	5'	87	**LYS**	**629**	C	20	60
**ARG**	**444**	**DA**	**26**	5'	100	**ASP**	**631**	C	20	94
**ARG**	**444**	**DC**	**25**	5'	50					
**LYS**	**448**	**DC**	**18**	3'	70					
**SER**	**450**	**DC**	**27**	5'	99					
**SER**	**450**	**DA**	**26**	5'	50					
**VAL**	**457**	**DC**	**27**	5'	88					
**LYS**	**459**	**DG**	**12**	3'	42					
					**orc1b**					
**ARG**	**100**	**DG**	**14**	3'	100	**ARG**	**315**	**C**	**7**	100
**HIS**	**101**	**DC**	**29**	5'	76	**ARG**	**321**	**C**	**11**	86
**LYS**	**106**	**DC**	**13**	3'	100	**ARG**	**378**	**G**	**13**	100
**ARG**	**315**	**DA**	**26**	5'	100	**ARG**	**381**	**G**	**13**	100
**ARG**	**315**	**DC**	**27**	5'	100	**ARG**	**437**	**C**	**20**	96
**GLN**	**369**	**DG**	**28**	5'	76	**ARG**	**830**	**C**	**9**	47
**ARG**	**381**	**DC**	**30**	5'	100	**SER**	**837**	**C**	**8**	56
**GLU**	**409**	**DC**	**30**	5'	88					
**ARG**	**830**	**DC**	**18**	3'	100					
**ARG**	**838**	**DC**	**25**	5'	100					
**ARG**	**838**	**DA**	**26**	5'	100					
**ARG**	**844**	**DG**	**20**	3'	100					
**ARG**	**846**	**DG**	**19**	3'	93					

Table S2.

Table 2. Percentage of occurrence of ligand-protein connections along the last half MD trajectory frames, for each atomic pair. Only the connections occurring in more than 40% of the frames are reported.

**dRM9**								**RM9**						
**orc1a**														
**ARG**	**19**	NH1/2	**DC**	**13**	3'	O1/2P	***67***	**ARG**	**297**	NH1/2	**C**	**3**	O2P	***100***
**ARG**	**191**	NH1/2	**DG**	**23**	5'	O1/2P	***100***	**ARG**	**437**	NH1/2/NE	**C**	**19**	*O2*	***61***
**SER**	**199**	OG	**DC**	**24**	5'	O1/2P	***51***	**SER**	**450**	N/OG	**C**	**5**	O1/2P	***100***
**SER**	**201**	N	**DC**	**24**	5'	O2P	***45***	**HIS**	**452**	NE2	**G**	**13**	O1P	***59***
**TYR**	**634**	OH	**DC**	**17**	5'	O1P	***59***	**THR**	**453**	OG1	**G**	**13**	O2P	***59***
**LYS**	**643**	NZ	**DC**	**19**	5'	O1/2P	***75***	**GLU**	**575**	OE1/2	**C**	**7**	*N4*	***75***
**ARG**	**666**	NE/NH2	**DC**	**17**	5'	O1/2P	***100***	**HIS**	**577**	NE2	**C**	**15**	O2'/O2	***50***
**SER**	**668**	OG	**DG**	**28**	3'	O1P	***67***	**GLN**	**578**	OE1	**C**	**15**	O2'	***55***
**ARG**	**670**	NH1/2	**DC**	**18**	5'	O1P	***100***	**LYS**	**591**	NZ	**G**	**2**	*O6/N7*	***57***
**LEU**	**673**	N	**DC**	**27**	3'	O1P	***44***	**THR**	**593**	OG1	**C**	**17**	O1P	***78***
**ARG**	**675**	NH1/2	**DG**	**28**	3'	O1/2P	***100***	**ARG**	**846**	NH1/2	**C**	**12**	O1/2P	***100***
**orc1b**														
**ARG**	**24**	NH1/2	**DG**	**23**	5'	*N7*	***66***	ARG	**378**	NH1/2	C	11	O1P	***95***
**LYS**	**25**	NZ	**DG**	**17**	3'	*O6/N7*	***62***	ARG	**378**	NH1/2	A	10	O2P	***65***
**SER**	**170**	OG/N	**DC**	**27**	3'	O1P	***56***	ILE	**379**	O	C	11	*N4*	***95***
**LYS**	**276**	NZ	**DC**	**29**	5'	O1/2P	***72***	ILE	**379**	N	C	11	*N3/O2*	***79***
**ARG**	**277**	NH1/2	**DT**	**15**	3'	O3'/O1P	***66***	LYS	**382**	NZ/N	C	7	O1/2P	***100***
**LEU**	**301**	N	**DG**	**28**	5'	O2P/O5'	***100***	SER	**383**	N/OG	C	8	O2P	***100***
**SER**	**302**	N	**DG**	**28**	5'	O2P	***99***	ARG	**433**	NH1/2/NE	C	5	O2P	***94***
**TYR**	**303**	N	**DG**	**28**	5'	O2P	***95***	ARG	**433**	NH1/2	G	6	O1/2P	***83***
**ARG**	**433**	NH1/2	**DC**	**25**	5'	O1P	***87***	ARG	**444**	NH1/2	C	5	O1/2P	***100***
**ARG**	**444**	NH1/2	**DA**	**26**	5'	O1P	***100***	ARG	**444**	NH1	G	4	O2P	***71***
**ARG**	**444**	NH1	**DC**	**25**	5'	O3'	***50***	LYS	**448**	NZ	G	2	O2'/O3'	***45***

**LYS**	**448**	NZ	**DC**	**18**	3'	O1P	***70***	SER	**450**	O	C	3	O2'	***60***
**SER**	**450**	OG	**DC**	**27**	5'	O1P	***99***	LYS	**629**	NZ	C	20	*O2/N3*	***60***
**SER**	**450**	N	**DA**	**26**	5'	O1P	***50***	ASP	**631**	OD1/2	C	20	O3'	***94***
**VAL**	**457**	N	**DC**	**27**	5'	O2P	***88***							

orc1c

**ARG**	**100**	NH1/2/NE	**DG**	**14**	3'	O1P	***100***	**ARG**	**315**	NH1/2	**C**	**7**	O1/2P	***100***
**HIS**	**101**	NE2	**DC**	**29**	5'	O3'	***76***	**ARG**	**321**	NH1/2	**C**	**11**	O1P	***73***
**LYS**	**106**	NZ	**DC**	**13**	3'	O1P/O5'	***98***	**ARG**	**378**	NH1/2/NE	**G**	**13**	*O6/N7*	***100***
**LYS**	**106**	NZ	**DC**	**13**	3'	O5'	***44***	**ARG**	**381**	NH2/NE	**G**	**13**	O1/2P	***100***
**ARG**	**315**	NH1/2	**DA**	**26**	5'	O1P	***100***	**ARG**	**437**	NE/NH2	**C**	**20**	*O2*	***96***
**ARG**	**315**	NH2/NE	**DC**	**27**	5'	O1P	***100***	**ARG**	**830**	NH1/2	**C**	**9**	O1P	***47***
**GLN**	**369**	NE2	**DG**	**28**	5'	O2P	***76***	**SER**	**837**	N	**C**	**8**	*O2*	***56***
**ARG**	**381**	NH1/2	**DC**	**30**	5'	O2P	***100***							
**ARG**	**381**	N	**DC**	**30**	5'	*O2/N3*	***81***							
**GLU**	**409**	O	**DC**	**30**	5'	O3'	***88***							
**ARG**	**830**	NH1/2	**DC**	**18**	3'	O1/2P	***100***							
**ARG**	**838**	NH1/2	**DC**	**25**	5'	O1/2P	***100***							
**ARG**	**838**	NH1	**DA**	**26**	5'	O2P	***96***							
**ARG**	**838**	N	**DA**	**26**	5'	O2P	***65***							
**ARG**	**838**	NH1	**DC**	**25**	5'	O5'	***42***							
**ARG**	**844**	NH1/2	**DG**	**20**	3'	O1/2P	***100***							
**ARG**	**846**	NH2	**DG**	**19**	3'	O1/2P	***93***							
